# Cu^II^ Ion Doping Enhances the Water Stability of Luminescent Metal–Organic Framework, Realizing the Detection of Fe^3+^ and Antibiotics in Aqueous Solutions

**DOI:** 10.3389/fchem.2022.860232

**Published:** 2022-02-28

**Authors:** Ruo-Qin Jia, Geng Tan, Ying-Jun Chen, Lu-Yang Zuo, Bo Li, Li-Ya Wang

**Affiliations:** College of Chemistry and Pharmacy Engineering, Nanyang Normal University, Nanyang, China

**Keywords:** Cu(II) ion doping, luminescent metal–organic frameworks, detection of Fe 3+, detection of antibiotics, Zn-MOF

## Abstract

Luminescent metal–organic frameworks (LMOFs) have been widely developed in the field of chemical sensing owing to their outstanding photoluminescence performance, high selectivity, anti-interference, high sensitivity, and fast response, and have become one of the research hotspots of emerging functional materials. However, in practical applications, many tests are carried out in the water environment, and fragile water stability greatly limits the application of MOFs in the field. Therefore, it is important to develop a method to enhance the water stability of MOFs. Herein, a new complex {[Zn(L)]·CH_3_CN}_*n*_ (**Zn-MOF**, H_2_L = 5-(benzimidazol-1-yl) isophthalic acid) with a superior photophysical property has been synthesized first. Its water stability was highly enhanced by the doping of Cu^II^ ions by the one-pot method. In addition, the detection performances of doping material Cu_0.1_/Zn-MOF for sixteen metal ions and thirteen antibiotics were well studied. It was found that Cu_0.1_/Zn-MOF displays high sensitivity, fast response, lower detection limit, and long-term stability for the detection of Fe^3+^, NFT, NFZ, FZD, and TC in the aqueous medium.

## Introduction

Antibiotics play important roles in the treatment of bacterial infections, but in recent decades, the overuse and even abuse of antibiotics have brought serious impacts on human health and ecological balance (Li et al., 2020; Zhong et al., 2020). On the other hand, iron, a necessary trace element of the human body, is essential to life activities, but Fe^3+^ ion, a kind of high-charge metal ion, usually causes environmental pollution and harms the health of life (Zhao et al., 2017; Panda et al., 2021). To ensure the health and safety of the ecosystem, it is necessary to establish an effective detection method for antibiotics and cations in the environment.

LMOFs have attracted extensive attention in the field of chemical sensing because of their excellent electronic and optical properties, designable main structure, porosity, fast response, and high sensitivity (Cui et al., 2012; Kreno et al., 2012; Yang et al., 2019; Esrafili et al., 2020; Hu et al., 2020; Yang et al., 2020a; Yang et al., 2020b; Esrafili et al., 2021). Some LMOFs have been used for the detection of antibiotics and cations, and have shown good detection performance and sensitivity (Zhao et al., 2017; Zhang et al., 2017b; Yi et al., 2018; Abdollahi et al., 2020; Li et al., 2021; Xiao et al., 2021). However, most tests can only be performed in organic solvents due to the fragile water stability of LMOFs. Therefore, the water stability of MOFs largely determines whether it can be further commercialized and applied. In this case, it is of great significance to develop a method that not only enhances the water stability of LMOFs but also ensures its detection performance.

At present, a variety of methods have been reported to improve the water stability of MOFs, but most of them focus on post-synthesis exchange (Liu et al., 2014; Yang et al., 2014), post-synthesis modification (Garibay and Cohen, 2010; Volkringer and Cohen, 2010), hydrophobic surface treatment (Hou et al., 2016; Qian et al., 2017), and composite hydrophobic materials (Yang and Park, 2012; Zhang et al., 2014). However, MOFs treated by the aforementioned methods are difficult to produce hydrophilic groups, which may not be conducive to practical industrial applications (Qiu et al., 2020). The strategy of doping metal ions into MOFs to improve water stability has been reported, and this approach may provide a new way to solve the stability problem (Li et al., 2012; Zhu et al., 2016). Li’s group improved the water stability of MOF-5 by doping Ni(II) for the first time using the thermal solvent method (Li et al., 2012). The result showed that the doping of Ni(II) ion not only improved the stability in the aqueous solution due to the formation of Ni_x_Zn_4-x_O^6+^ secondary construction unit, but also increased the specific surface area and pore size of MOF-5. Zhu’s group doped metal ions Cu^2+^, Fe^2+^, and Cd^2+^ into STU-1 with poor hydrostability (Zhu et al., 2016). After doping metal ions, the crystallinity remained well and their structures remained unchanged after being soaked in boiling water for 7 days. The water adsorption isotherm indicated that the STU-1s doped with metal ions was a strongly hydrophobic material. The authors speculated that the enhanced hydrophobicity may be ascribed to the disturbance of the doped metal ions on the surface of MOFs, which hindered the formation of water clusters. Wang’s group synthesized MIL-101(Cr) doped with Ni ion by presynthesis method for the first time (Qiu et al., 2020). The result suggested that the Ni-doped MIL-101(Cr) retains its octahedral shape, high specific surface area, and large pore size. The stability of MIL-101(Cr) with Ni doping is significantly improved in various pH environments. This strategy of doping inert metal ions is significant for the practical application of LMOFs. First, the enhancement of water stability after doping makes it possible for cycle detection in the water environment. Second, the hydrophilic structure of the original MOFs is retained after doping, which is more conducive to its efficient operation in the water environment (Qiu et al., 2020).

In this work, {[Zn(L)]·CH_3_CN}_*n*_ (**Zn-MOF**) was synthesized using Zn(NO_3_)_2_·6H_2_O and 5-(benzimidazol-1-yl) isophthalic acid (H_2_L) ligand. The selection of H_2_L ligand is based on the following considerations: 1) H_2_L ligand containing carboxylic acid groups, aromatic ring, and benzimidazol-1-yl fluorescence conjugated groups is beneficial to the formation of large Stokes shift, which may have potential in fluorescence detection (Zhang et al., 2017a; Liu et al., 2018; Park et al., 2020) and the sensitivity (Zhou et al., 2018); 2) the various coordination modes of carboxylic acids increase the coordination diversity of complexes (Fan et al., 2021); 3) the flexible benzimidazol-1-yl arm can fine-tune the coordination structure through axial rotation (Cheng and Kuai, 2012). Although subsequent experiments displayed that **Zn-MOF** had good fluorescence intensity in the water environment, their frames gradually disintegrated, which was also confirmed by the changes of powder X-ray diffraction (PXRD) and emission wavelength after immersion in aqueous solutions for 7 days. Therefore, in order to realize the recycling of **Zn-MOF** as sensors in the water environment, it is necessary to improve its hydrostability without losing its detection performance. The method of doping inert metal ions into the existing skeleton was considered first. Considering that **Zn-MOF** has a binuclear paddle-wheel secondary building unit (SBU), we chose Cu(II) ion as the pre-doped metal ion. The main considerations are as follows: 1) The metal ions in binuclear paddle-wheel SBUs with D_4h_ being symmetric can be Cu^II^, Zn^II^, Co^II^, Fe^II^, Cd^II^, *etc*., but Cu^II^-paddle wheel SBUs generally have better thermodynamic stability (Song et al., 2012; Wei et al., 2013). 2) In previous reports, Cu^II^ ions have been successfully incorporated into Zn^II^ paddle-wheel binuclear clusters (Zhang et al., 2011; Song et al., 2012; Wei et al., 2013). 3) Fluorescence materials with large Stokes shift will be an interesting topic. Doping Cu ions into the main frame may alter the optical properties of the materials, providing greater Stokes shifts (Liu et al., 2015). Fortunately, we directly synthesized a series of Cu/Zn bimetallic MOFs with different Cu ion doping ratios by the one-pot method. The water stability of the doped MOFs is significantly higher than that of the original **Zn-MOF**. Subsequently, Cu_0.1_/Zn-MOF was selected as a fluorescence sensor for fluorescence detection of sixteen metal ions and thirteen antibiotics in aqueous solutions. The fluorescence detection results showed that Fe^3+^, NFT, NFZ, FZD, and TC had obvious fluorescence quenching for Cu_0.1_/Zn-MOF. It is noteworthy that the Cu^II^ ion doping strategy improves not only the water stability of the original LMOFs but also their detection sensitivity.

## Experimental

### Materials and General Methods

Starting reagents, solvents, and materials were commercially available and at least of analytical grade. The H_2_L ligands were purchased from Jinan Henghua Sci. and Tec. Co. Ltd. PXRD patterns at diffraction angles from 5° to 55° were obtained with a D/MAX-3D diffractometer. Elemental analysis was performed by Perkin–Elmer Elementarvario elemental analysis instrument. Fourier transform infrared spectra (FT-IR) were recorded on Nicolet iS50 (4,000–400 cm^−1^). Thermogravimetric analysis (TGA) was performed on a SDT 2960 thermal analyzer from room temperature to 800°C at a heating rate of 10°C/min under nitrogen flow. Ultraviolet-visible (UV-vis) absorption spectra were obtained by UV-2600 UV-vis spectrophotometer. Fluorescence detection was carried out on CARY Eclipse Fluorescence Spectrophotometer at room temperature. Energy dispersive spectrometer (EDS) was obtained by JSM-6490LV (JEOL Ltd., Japan) electron microscope.

### X-Ray Crystallographic Analysis

Single-crystal X-ray diffraction data of **Zn-MOF** were collected by Oxford Diffraction SuperNova area-detector diffractometer with the program of CrysAlisPro. The crystal structure was solved by SHELXS-2016 and SHELXL-2016 software (Sheldrick 2008). The crystallographic data and structure refinements are shown in Table 1. The CIF file of **Zn-MOF** (CCDC No. 2143524) can be downloaded free of charge *via* http://www.ccdc.cam.ac.uk/conts/retrieving.html. Selected bond lengths and bond angles for the **Zn-MOF** complex are shown in Supplementary Table S1.

**TABLE 1:** Crystallographic data for Zn-MOF complexes.

Crystal data	Zn-MOF
Formula	C_17_H_11_N_3_O_4_Zn
Formula weight	386.66
Crystal system	Monoclinic
Space group	*P*2_1_ */c*
*a*/Å	10.8973(4)
*b*/Å	10.2359(3)
*c*/Å	15.6463(5)
*α*/°	90
*β*/°	108.863(4)
*γ*/°	90
Volume/Å^3^	1707.33(15)
*Z*	4
Dcalc (g cm^−3^)	1.504
Absorption coefficient (mm^−1^)	1.465
*F*(000)	784
*R*_int_	0.0283
GOF on *F* ^2^	1.008
R indices [*I*>2σ(*I*)]	R_1_ = 0.0376, wR_2_ = 0.1019
R indices (all data)	R_1_ = 0.0476, wR_2_ = 0.1082

### Water Stability Test

Powder samples (120 mg) were dispersed in vials containing 10 ml of aqueous solution, respectively. The vials were kept at room temperature for 1–7 days, and 30 mg samples were taken out at 1, 3, and 7 days, respectively, for PXRD tests. The results demonstrated that the doping of Cu ions improved the water stability of the **Zn-MOF**. In order to investigate the stability, Cu_0.1_/Zn-MOF powder was dispersed in a vial containing 3 ml aqueous solutions with different pH scales (pH = 1–14) for 1 h, and then the PXRD test was carried out. In addition, in order to explore the fluorescence performance of Cu^2+^ doped **Zn-MOF** at different pH values, the synthesized sample Cu_0.1_/Zn-MOF (2.0 mg) was added into aqueous solutions (3.0 ml) with different pH values, and ultrasonicated for 20 min; then the fluorescence spectra were immediately determined.

### Luminescence Sensing Experiments

For the detection of metal ions, 2.0 mg samples were finely ground and added into 3.0 ml of deionized water of M(NO_3_)_x_ (1 mM, M = Na^+^, Ca^2+^, K^+^, Li^+^, Zn^2+^, Mg^2+^, Co^2+^, Mn^2+^, Cd^2+^, Ni^2+^, Ag^+^, Pb^2+^, Cu^2+^, Al^3+^, Cr^3+^, and Fe^3+^). For the detection of antibiotics, 2.0 mg samples were finely ground and added into 3.0 ml of varied selected antibiotics solutions (100 ppm), including tetracyclines (tetracycline TC; nystatin NS), sulfonamides (sulfamethoxydiazine SM), chloramphenicols (chloramphenicol CHL), aminoglycosides (gentamicin GEN; kanamycin KAN; hygromycin HYG; streptomycin sulfate STR; spectinomycin SH), nitrofurans (nitrofurazone NFZ; nitrofurantoin NFT; furazolidone FZD), and β-lactams (cefotaxime CEF). In order to maintain its homogeneity, the mixtures were ultrasonicated for 20 min to form a suspension. The luminescence data of the suspension were monitored under the same conditions.

### Synthesis of Zn-MOF

A mixture of Zn(NO_3_)_2_·6H_2_O (29.8 mg, 0.1 mmol), H_2_L (14.1 mg, 0.05 mmol), CH_3_CN/H_2_O (2 ml/3 ml), and one drop of dilute HNO_3_ (1 M) was added to a 10 ml Pyrex vial and stirred for 20 min. Then it was transferred to a Teflon-lined stainless steel vessel and reacted at 120°C for 72 h. After being cooled to room temperature at a rate of 5°C min^−1^, the light yellow block crystals were obtained by filtration and collection, washed with CH_3_CN, and then air-dried naturally with a yield of 83.8% (based on H_2_L. Anal. Calc. (%) for C_17_H_11_N_3_O_4_Zn (Mr = 386.68): C 52.80 H 2.87, N 10.87; found (%): C 52.85, H 2.91, N 10.82. IR (KBr pellet, cm^−1^): 3,468 (w), 3,105 (m), 3,074 (w), 2,246 (w), 1837 (w), 1786 (w), 1,654 (s), 1,589 (s), 1,511 (s), 1,458 (s), 1,426 (s), 1,388 (s), 1,310 (s), 1,239 (s), 1,179 (m), 1,112 (m), 1,043 (w), 1,004 (w), 921 (s), 850 (w), 784 (s), 750 (s), 718 (s), 689 (m), 648 (w), 531 (m), 456 (s).

**Synthesis of Cu**_**x**_ **/Zn-MOF (x = 0.01, 0.1, 0.2, 0.5)**: A mixture of Zn(NO_3_)_2_·6H_2_O (29.5 mg, 0.099 mmol; 26.8 mg, 0.09 mmol; 23.8 mg, 0.08 mmol; 14.9 mg, 0.05 mmol), Cu(NO_3_)_2_·3H_2_O (0.242 mg, 0.001 mmol; 2.42 mg, 0.01 mmol; 4.83 mg, 0.02mmol; 12.1 mg, 0.05 mmol), H_2_L (14.1 mg, 0.05 mmol), CH_3_CN/H_2_O (2 ml/3 ml), and two drops of HNO_3_ (62%, aq.) was added to a 10 ml Pyrex vial and stirred for 20 min, and then transferred to a Teflon-lined stainless steel vessel and reacted at 120°C for 72 h. After being cooled to room temperature at a rate of 5°C min^−1^, the light blue block crystals were obtained by filtration and collection, washed with CH_3_CN, and then air-dried naturally with a yield of 81.2, 82.9, 81.1, and 82.7%, respectively (based on H_2_L).

## Results and Discussion

### Crystal Structure of {[Zn(L)]·CH_3_CN}_*n*_ (Zn-MOF)

**FIGURE 1:**
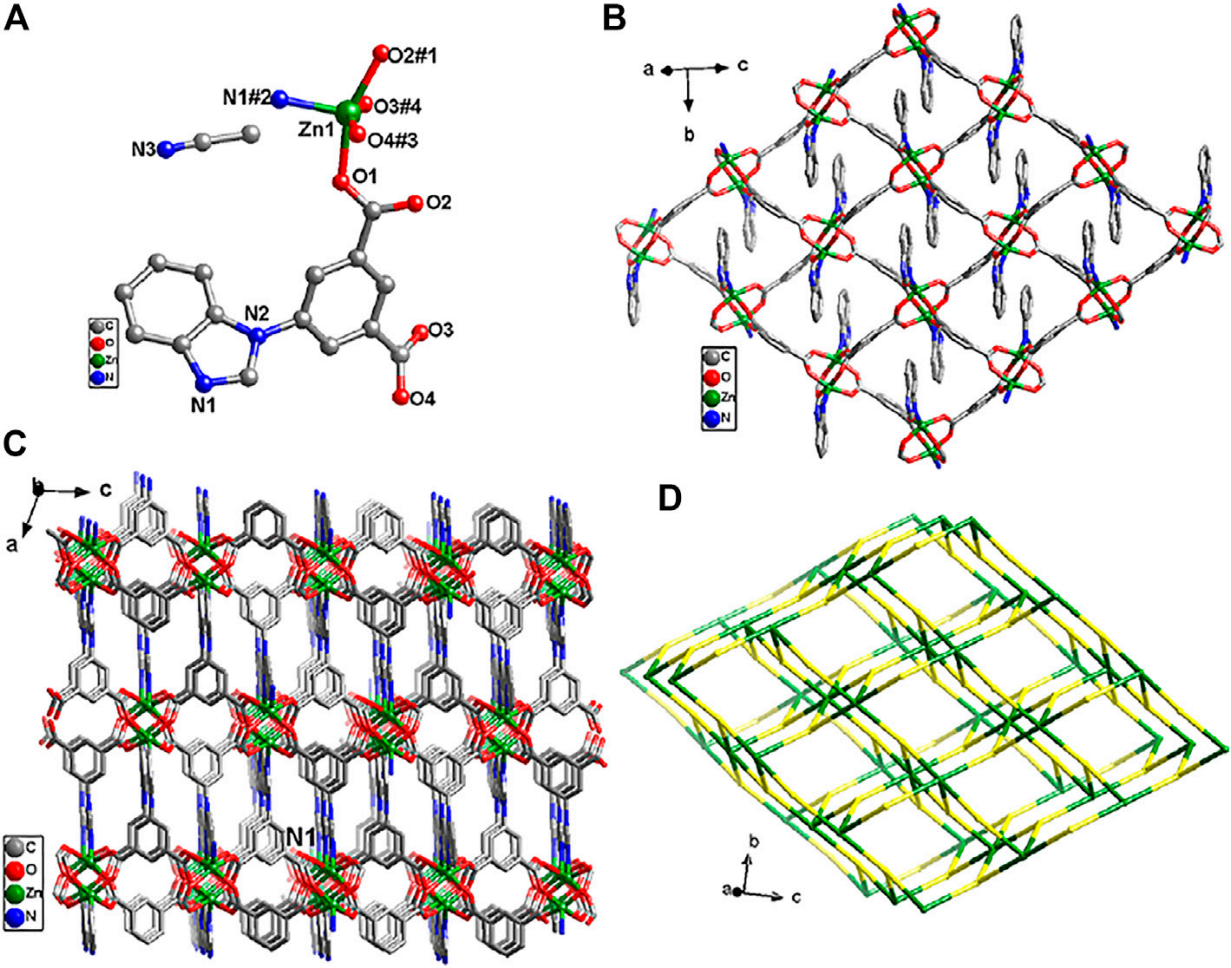
**(A)** Coordination environment of Zn^2+^ ion. **(B)** The 2D layered structure of Zn-MOF. **(C)** View of the three-dimensional structure of Zn-MOF. **(D)** Topological analysis of Zn-MOF. Symmetry codes: #1 −x+1, −y+1, −z; #2 −x+2, y+1/2, −z+1/2; #3 x, −y+1/2, z−1/2; #4 −x+1, y+1/2, −z+1/2.

Single-crystal X-ray analysis reveals that {[Zn(L)]·CH_3_CN}_*n*_ crystallizes in the monoclinic system, *P*2_1_/*c* space group. In **Zn-MOF**, the asymmetric unit contains one Zn(II) atom, one fully deprotonated L^2-^ ligand, and one free acetonitrile molecule (Figure 1A). Zn(II) atom is in the five-coordination mode with four oxygen atoms from four carboxylic acid ligands and one nitrogen atom from the benzimidazolium. Zn–O bond lengths range from 2.027(2) to 2.035(2) Å, and Zn–N bond length is 2.021(2) Å. Adjacent two zinc atoms were bridged by four carboxyl groups to form a bimetallic unit. Each bimetallic unit was connected with four nodes in four directions by different isophthalate groups to generate an infinite 2D layer (Figure 1B). Adjacent layers were joined by nitrogen atoms (N1) of imidazole groups to give rise to a three-dimensional structure (Figure 1C). From a topological viewpoint, each dinuclear Zn(II) cluster connects six L^2-^ ligands, and each L^2-^ ligand links three dinuclear Zn(II) clusters. As illustrated in Figure 1D, **Zn-MOF** could be simplified as a 3,6-c network with the Schläfli symbol {4·6^2^}_2_{4^2^·6^10^·8^3^} with TOPOS (Blatov et al., 2014).

### EDS and FTIR

The chemical compositions of **Zn-MOF** and Cu_x_/Zn-MOF were determined by EDS (Supplementary Figures S1–S5), as displayed in Supplementary Table S2. The results showed that the Cu/Zn ratios were obviously higher than the synthesis ratio. This phenomenon may be due to stronger bonds formed between Cu(II) ions and H_2_L ligands (Niu et al., 2014). The SEM-mapping of the Cu_0.1_/Zn-MOF shown in Supplementary Figure S6 further indicates the existence of copper and the uniform distribution of copper and zinc in the skeleton structure.

The FTIR spectra of **Zn-MOF** and Cu_x_/Zn-MOF (x = 0.01, 0.1, 0.2, 0.5) were carried out and shown in Supplementary Figure S7. The C=O stretching vibration of the free ligand H_2_L is 1720 cm^−1^, while it is not visible in the five complexes, which illustrates the coordination of carboxylic acids with metals. The vibration band at 1,589 cm^−1^ and 1,389 cm^−1^ of the five complexes can be associated with the asymmetrical stretching vibration and symmetrical stretching vibration of -COO-, respectively. The observed band at 1,653 cm^−1^ in the FTIR spectrum of Zn-MOF is assigned as the stretching vibration of the -C=N- bond (Huang et al., 2020), which migrates to the lower band with Cu ion doping (Cu_0.01_/Zn-MOF: 1,653 cm^−1^, Cu_0.1_/Zn-MOF: 1,651 cm^−1^, Cu_0.2_/Zn-MOF: 1,647 cm^−1^, Cu_0.5_/Zn-MOF: 1,639 cm^−1^), probably due to competitive coordination between Cu^2+^ and Zn^2+^. The signals at 455 cm^−1^ can be attributed to the stretching vibration of Zn–O. For the copper-doped **Zn-MOF**, slight shifts (Cu_0.01_/Zn-MOF: 455 cm^−1^, Cu_0.1_/Zn-MOF: 461 cm^−1^, Cu_0.2_/Zn-MOF: 467 cm^−1^, Cu_0.5_/Zn-MOF: 478 cm^−1^) were observed, suggesting the coordination of Cu with the carboxylic group of H_2_L ligand. The FTIR spectra of **Zn-MOF** and Cu-doped **Zn-MOF** are almost the same, which further indicates that the two materials exhibit an isomorphic structure.

### Thermal and Chemical Stability

The thermal stabilities of compounds Zn-MOF and Cu_x_/Zn-MOF (x = 0.01, 0.1, 0.2, 0.5) were examined by TGA under a N_2_ atmosphere in the temperature range 25–800°C. As shown in Supplementary Figure S8, in the **Zn-MOF** complex, the weight loss rate reached 9.82% (calcd.10.61%) at 152–275°C, which is to the loss of one acetonitrile molecule. Then the skeleton of the compound began to collapse at 419 °C. The analysis of TGA curve of Cu_0.01_/Zn-MOF confirmed that the weight loss rate was 10.34% (calcd.10.61%) at 167–275°C, and the skeleton of the compound began to collapse at 413°C. For Cu_0.1_/Zn-MOF, the weight loss rate was 10.22% (calcd.10.61%) at 166–300°C, and the skeleton of the compound began to collapse at 381°C. For Cu_0.2_/Zn-MOF, the weight loss rate was 9.31% (calcd.10.61%) at 167–295°C, and the skeleton of the compound began to collapse at 352°C. For Cu_0.5_/Zn-MOF, the weight loss rate was 9.27% (calcd.10.61%) at 170–295°C, and the skeleton of the compound began to collapse at 348°C. We found that the doping of Cu(II) ions have a significant effect on the decomposition temperature of organic frames. With the increase of the Cu(II) ion doping ratio, the thermal stability decreases gradually, which probably due to copper divalent is easier to be reduced than zinc divalent at high temperature.

**FIGURE 2:**
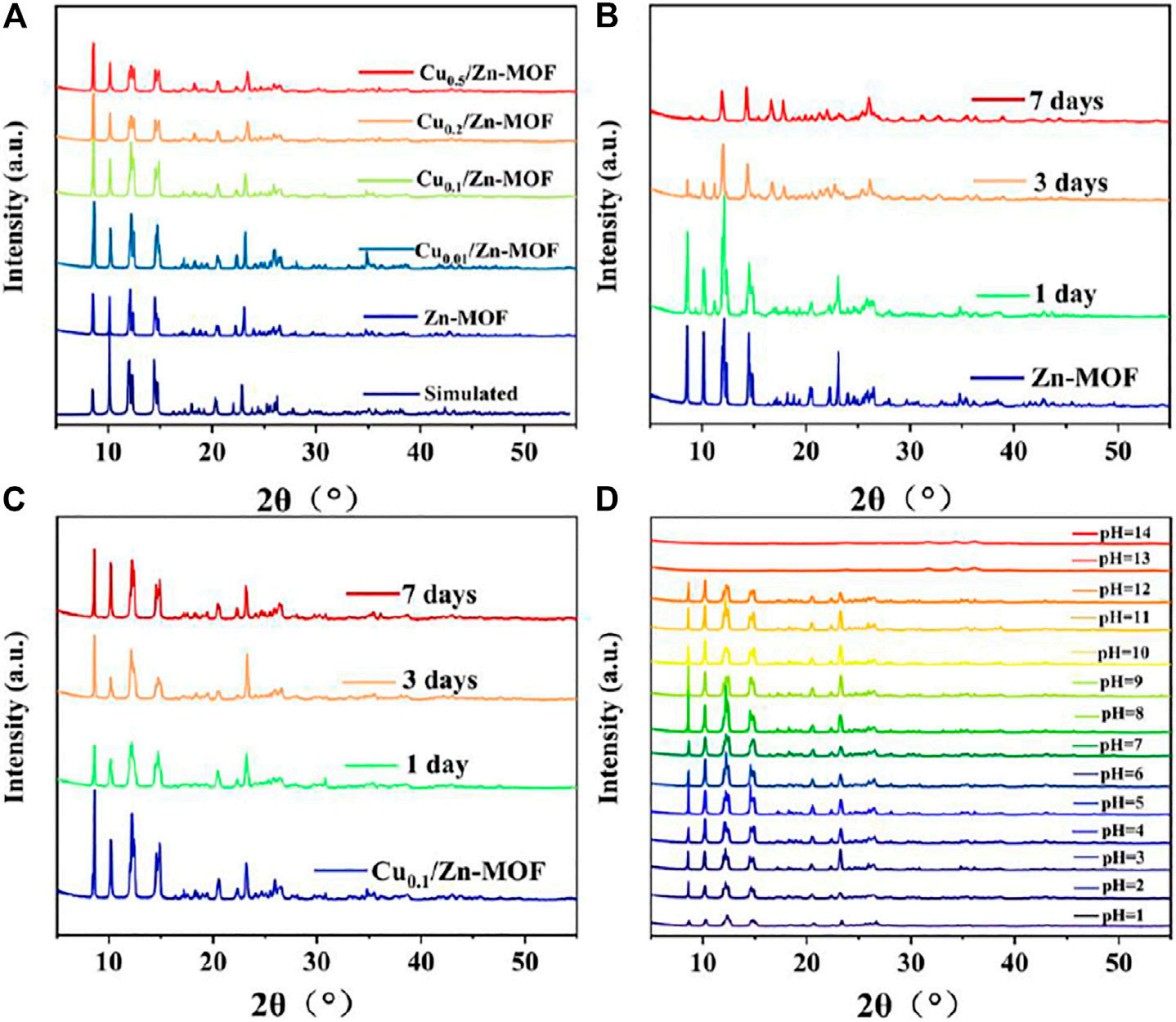
**(A)** PXRD patterns of **Zn-MOF** and Cu_x_/Zn-MOF (x = 0.01, 0.1, 0.2, and 0.5). PXRD patterns of Zn-MOF **(B)** and Cu_0.1_/Zn-MOF **(C)** soaked in water for 1, 3, and 7 days, respectively. **(D)** PXRD patterns of Cu_0.1_/Zn-MOF soaked in different pH (1–14) solutions for 1 h.

As shown in Figure 2A, the PXRD spectra of **Zn-MOF** were in good agreement with the simulation diagram, indicating the high degree of pure phase. When different proportions of Cu^II^ were doped into **Zn-MOF**, the positions of diffraction peaks were unchanged, suggesting that copper doping did not change the crystal structure of **Zn-MOF**. The doped Cu ion may be incorporated into the framework of **Zn-MOF** (Cao et al., 2018).

In order to test the stability of the skeleton structure, the **Zn-MOF** powder sample was soaked in water for 7 days, and its PXRD patterns were investigated. As shown in Figure 2B, after soaking in water for one day, the **Zn-MOF** sample remained basically crystalline, but the peak value was weakened, and an additional peak appeared at 2θ = 11.2°, indicating that the frame began to decompose (Kaye et al., 2007). The peak value was significantly reduced at 3 days, showing an acceleration of decomposition. After one week, the structure changed and the skeleton became unknown. As shown in Figure 2C and Supplementary Figure S9, four Cu-doped bimetal samples were immersed in water for 7 days, and their peak positions matched well, indicating that Cu-doped **Zn-MOF** had stronger water stability. The enhancement of water stability may be due to the formation of stronger coordination bonds after Cu^II^ replaces part of Zn^II^ in the original skeleton structure, which possibly improves the thermodynamic stability of metal clusters (Ding et al., 2019; Qiu et al., 2020). In practical applications, the chemical stability was also important in an aqueous environment. Therefore, the chemical stabilities of Cu_0.1_/Zn-MOF were studied by soaking samples in water with varying pH (from 1 to 14), and adjusted using HCl and NaOH. PXRD patterns showed that samples were highly resistant and matched well with the original sample in a pH range of 3–12 (Figure 2D).

### Fluorescence Properties

It is known that d^10^ configuration of Zn^II^ is difficult to oxidize or reduce; thus, the metal-to-ligand charge transfer (MLCT) or ligand-to-metal charge transfer (LMCT) is difficult to occur (Zhang et al., 2010). However, Zn^II^ ion coordinated with conjugated organic ligands may cause intraligand charge transfer (LLCT) (Yao et al., 2019). H_2_L ligand containing carboxylic acid and aromatic and N-containing heterocycle may exist in π*→π or/and π*→n electronic transitions.

The fluorescence emission of H_2_L ligand and various complexes in water were detected. As shown in Supplementary Figure S10, the fluorescence of **Zn-MOF** was significantly enhanced at 397 nm relative to the ligand in aqueous solution. The increased fluorescence may probably be attributed to LLCT induced by Zn^II^ ion (Dong et al., 2018), which may improve the rigidity of the structural unit due to the coordination interactions between the flexible ligand and Zn^II^, and finally lead to the reduction of the non-radiative relaxation of the excited state (Dong et al., 2019). Subsequently, with the increase of the Cu^II^ doping ratio, the emission intensity gradually decreases. When the Cu^II^ doping rate increases to 0.5, the fluorescence emission intensity is almost equal to that of H_2_L ligand. It is well known that the unsaturated electronic state of Cu^II^ ion (3d^9^) may cause MLCT, which causes the fluorescence quenching (Cui et al., 2012; Niu et al., 2014; Zheng et al., 2017). After doping of Cu^II^ ion, a new electron cloud distribution was formed, which may increase the energy loss of the system through non-radiative d-d transitions, and also weaken the LLCT, ultimately leading to a decrease in fluorescence intensity.

### Selection of Fluorescence Sensor

For a qualified fluorescence sensor, the stability of its frame structure, the luminescence intensity, and the sensitivity of fluorescence detection should be fully considered. Based on the aforementioned situation, Cu_0.1_/Zn-MOF was selected as the fluorescence sensor for subsequent fluorescence detection. Three factors were mainly taken into account: First, although **Zn-MOF** has stronger fluorescence intensity than Cu/Zn-MOF, its fragile structural stability in the water environment precludes its possibility of being used as a fluorescence sensor in the water, which can also be verified by its seven-day fluorescence spectra in water (Supplementary Figure S11). From the perspective of structural and fluorescence stability in water, Cu^II^ ion–doped **Zn-MOF** is a better choice (Supplementary Figures S10, S12). Second, in terms of fluorescence emission intensity, Cu_0.01_/Zn-MOF and Cu_0.1_/Zn-MOF are slightly weaker than **Zn-MOF**, but significantly stronger than Cu_0.2_/Zn-MOF and Cu_0.5_/Zn-MOF. Therefore, Cu_0.01_/Zn-MOF or Cu_0.1_/Zn-MOF may be more suitable as fluorescence sensors. Third, Stokes shift is also an important factor to consider. In fluorescence detection, the increase of Stokes shift is beneficial to reduce background interference and enhance the signal-to-noise ratio and detection sensitivity (Kundu et al., 2012; Ren et al., 2018). As shown in Supplementary Figure S10, compared with the Stokes shift of 114 nm (λ_ex_ = 283 nm, λ_em_ = 397 nm) of Cu_0.01_/Zn-MOF, the Stokes shift of Cu_0.1_/Zn-MOF is up to 140 nm (λ_ex_ = 257 nm, λ_em_ = 397 nm). As the fluorescence sensing material with large Stokes shift is rare and attractive, Cu_0.1_/Zn-MOF was selected as the fluorescence sensor for subsequent fluorescence detection.

Subsequently, the time-varying fluorescence spectra of Cu_0.1_/Zn-MOF samples in the water environment were measured. As shown in Supplementary Figure S13, the fluorescence intensity of Cu_0.1_/Zn-MOF in the water at different time periods did not change significantly. In addition, the fluorescence spectra of Cu_0.1_/Zn-MOF at different pH values were also measured. As shown in Supplementary Figure S14, the fluorescence emission spectra of Cu_0.1_/Zn-MOF at a pH range from 4 to 11 did not change significantly, which proved that the fluorescence of Cu_0.1_/Zn-MOF also has good acid–base stability.

### Detection of Metal Cations

**FIGURE 3:**
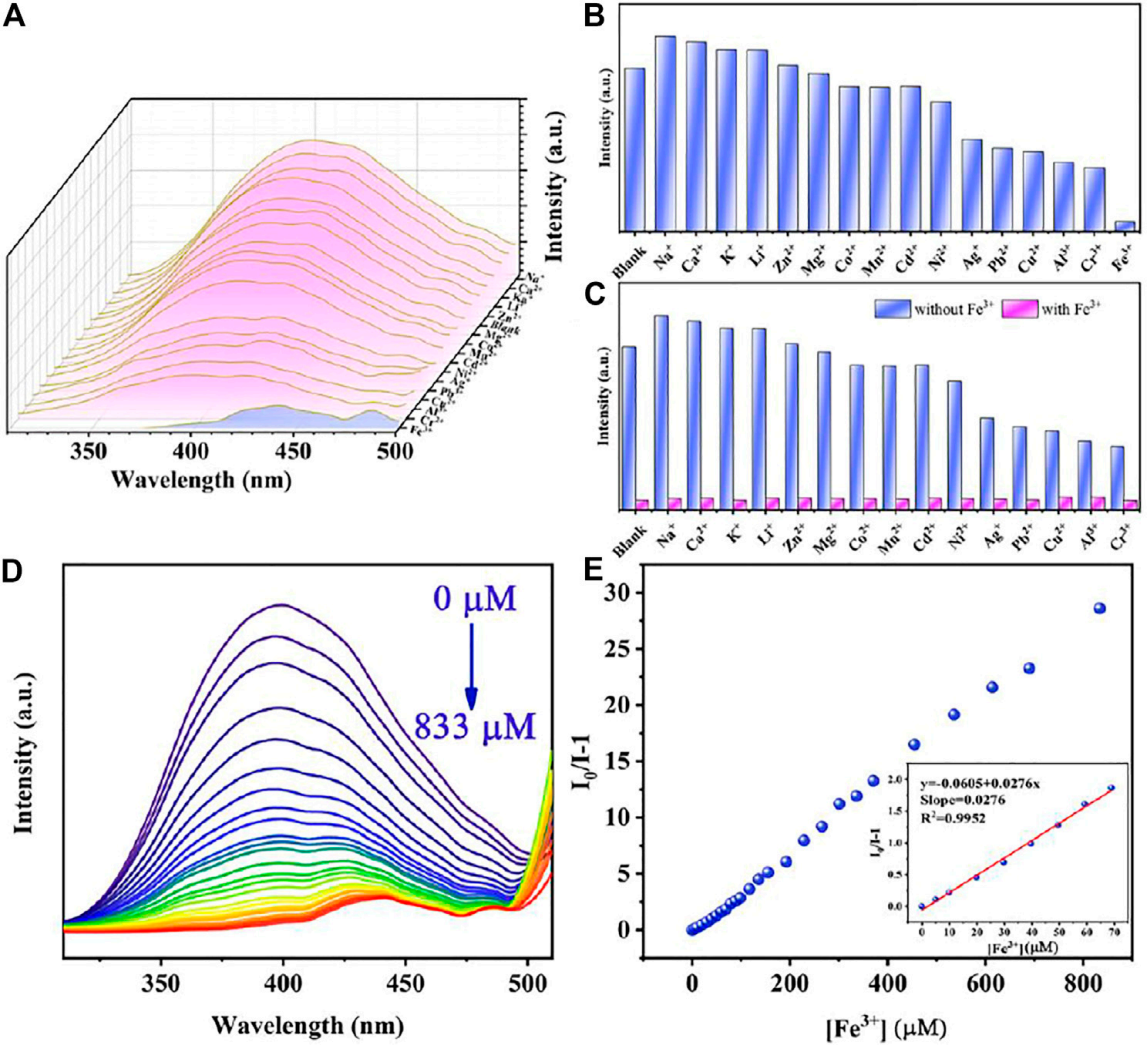
**(A)** Emission spectra of Cu_0.1_/Zn-MOF immersed in aqueous solutions with metal ions. **(B)** Sensing effects of Cu_0.1_/Zn-MOF on different metal ions. **(C)** Selective detection of Fe^3+^ on Cu_0.1_/Zn-MOF in the presence of different metal ions in aqueous solutions. **(D)** Photoluminescence spectra of Cu_0.1_/Zn-MOF in aqueous solutions upon incremental addition of Fe^3+^. **(E)** Stern–Volmer plot of Cu_0.1_/Zn-MOF upon adding different concentration of Fe^3+^.

For extensive purpose, sixteen metal ions, including Na^+^, Ca^2+^, K^+^, Li^+^, Zn^2+^, Mg^2+^, Co^2+^, Mn^2+^, Cd^2+^, Ni^2+^, Ag^+^, Pb^2+^, Cu^2+^, Al^3+^, Cr^3+^, and Fe^3+^ at the same concentration, were utilized for investigating the sensibility of Cu_0.1_/Zn-MOF. As shown in Figures 3A,B, compared with other cations, only Fe^3+^ ion showed obvious fluorescence quenching behavior. The fluorescence intensity at 397 nm was impaired by 93.8% compared with the initial value. This indicated that Cu_0.1_/Zn-MOF can be used as a visual sensor for selective detection of Fe^3+^ ion in water. The selectivity experiment of Cu_0.1_/Zn-MOF was explored. As shown in Figure 3C, the results showed that the interference of other cations can be ignored in the process of Fe^3+^ ion detection, which further confirms the selectivity of Cu_0.1_/Zn-MOF for Fe^3+^ detection.

In order to explore the limit of detection (LOD) of Cu_0.1_/Zn-MOF as a fluorescence sensor for the detection of Fe^3+^ ion, a fluorescence titration experiment was carried out. As shown in Figure 3D and Supplementary Figure S15, with the concentration of Fe^3+^ ion increased, the fluorescence intensity of the suspension decreased gradually, and the fluorescence quenching can be observed when the concentration of Fe^3+^ ion was 833 μM. The linear Stern–Volmer (S-V) equation, (I_0_/I) = K_SV_[C] + 1, can be used to explain the quenching efficiency (Li et al., 2016), where I_0_ and I represent the fluorescence intensities of Cu_0.1_/Zn-MOF suspension at 397 nm before and after addition of Fe^3+^, respectively. K_SV_ is the Stern–Volmer constant and [C] is the concentration of Fe^3+^. As shown in the Stern–Volmer plot of Cu_0.1_/Zn-MOF (Figure 3E) in the concentration range of 0–70 μM, the KSV was calculated to be 2.76 × 10^4^ M^−1^ (*R* ^2^ = 0.995). According to the slope and standard error of the fitting curve, the LOD for Fe^3+^ was calculated to be 0.76 μM according to 3σ/K_SV_ (Qu et al., 2020), where *σ* is the standard deviation for eleven repeated luminescent measurements (*σ* = 0.0070). The sensitivity of Cu_0.1_/Zn-MOF is a rival to most of the reported MOF-based sensors for Fe^3+^ (Supplementary Table S3), suggesting that it has potential application value in the detection of Fe^3+^.

The study of fluorescence quenching mechanism is of great significance for the exploration of more effective fluorescence materials. In the previous reports on the detection of metal ions, the quenching of fluorescence is usually caused by structure collapse, cation exchange, excitation energy competitive absorption, and energy resonance transfer (Mi et al., 2019). As shown in Supplementary Figure S9, after soaking in Fe^3+^ solution for 4 h, the PXRD patterns were unchanged, indicating that the fluorescence quenching is attributed to neither skeleton collapse nor cation exchange. In addition, the rapid response of Fe^3+^ detection also ruled out the possibility of fluorescence quenching caused by cation exchange because cation exchange cannot occur in a short period of time. As can be seen in Supplementary Figure S16, Fe^3+^ ions have a wide absorption band from 200 to 450 nm, which overlaps with the emission spectra range 310–510 nm of Cu_0.1_/Zn-MOF. Therefore, the main reason for fluorescence quenching may be on account of the fluorescence resonance energy transfer (FRET) (Nagarkar et al., 2013). In addition, Fe^3+^ ion has a higher UV absorption at 257 nm, and when the excitation wavelength chose 257 nm, Fe^3+^ ion has a higher excitation energy competitive absorption.

### Detection of Antibiotics

**FIGURE 4:**
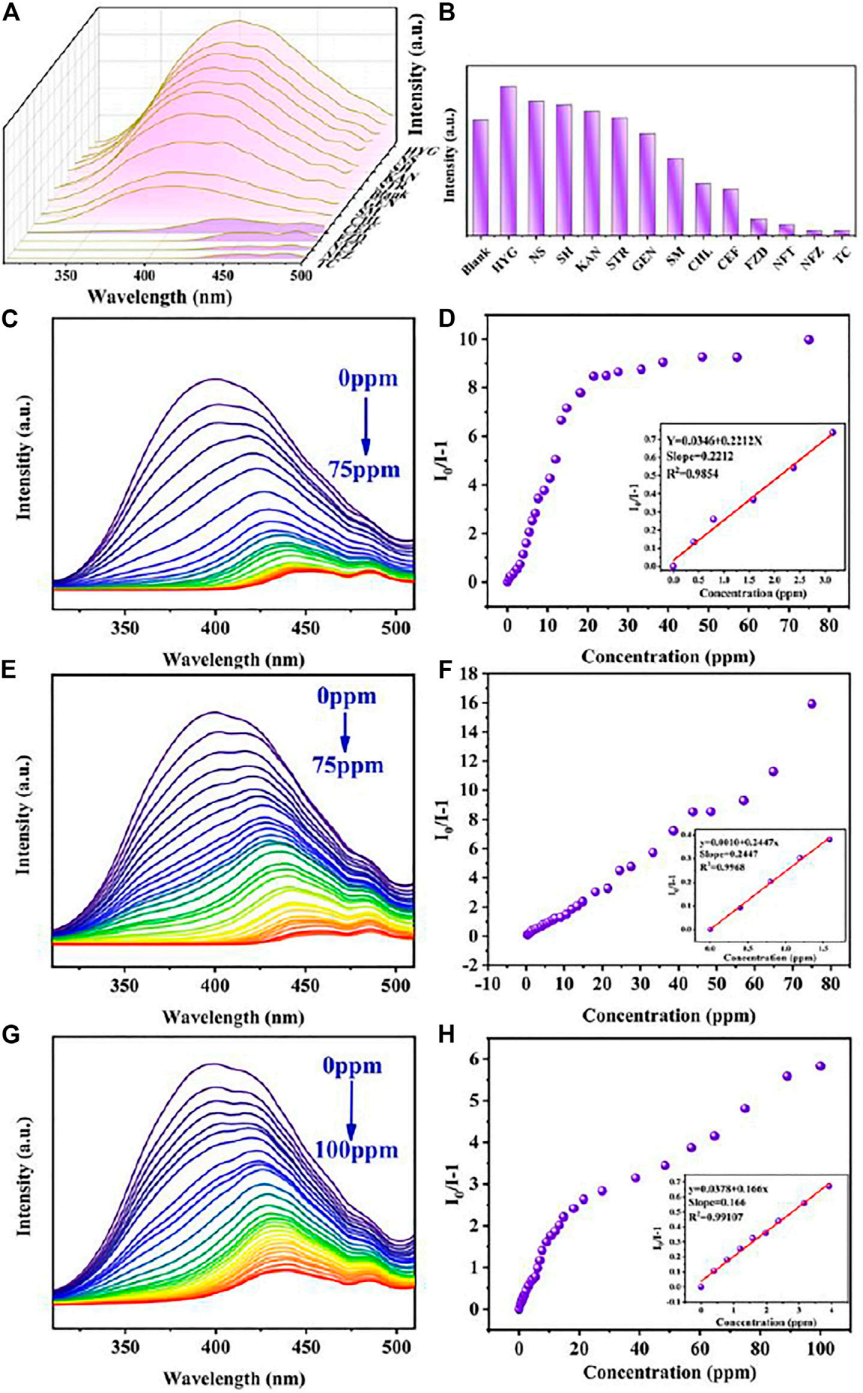
**(A)** Emission spectra of Cu_0.1_/Zn-MOF immersed in antibiotics. **(B)** Sensing effects of Cu_0.1_/Zn-MOF on different antibiotics. Photoluminescence spectra of Cu_0.1_/Zn-MOF in aqueous solutions upon incremental addition of NFT **(C)**, NFZ **(E)**, and FZD **(G)**. Stern–Volmer plot of Cu_0.1_/Zn-MOF upon adding different concentrations of NFT **(D)**, NFZ **(F)**, and FZD **(H)**.

Due to the excellent fluorescence performance and water stability of Cu_0.1_/Zn-MOF, the detection performances of Cu_0.1_/Zn-MOF on thirteen common antibiotics in six categories were studied. The results showed that NFT, NFZ, FZD, and TC had obvious quenching effect compared with other antibiotics (Figure 4).

### Luminescent Detection Toward Nitrofurans

In order to explore the sensitivity of Cu_0.1_/Zn-MOF as a fluorescence sensor for the detection of nitrofurans, fluorescence titration experiments were carried out. The detection of nitrofuran antibiotics mostly focused on fluorescence enhancement or quenching. In this experiment, with the increase of titrated concentration, the fluorescence emission peak not only showed fluorescence quenching (Supplementary Figure S17) but also displayed obvious redshift (NFT: 397–448 nm, NFZ: 397–456 nm, FZD: 397–440 nm) (Figure 4). Generally, the decrease of fluorescence intensity and the redshift of fluorescence emission peak with the increase of the detection concentrates are rare. The emission wavelength shift may be attributed to a strong interaction between the analyte and the sensor (Hu et al., 2013; Lei et al., 2021). To verify this interaction, three nitrofurans were added separately to Cu_0.1_/Zn-MOF solution. As shown in Supplementary Figure S18, the UV absorption peaks were found to be redshifted, which indicated the existence of static quenching, strong interactions, and the formation of ground state complexes (Zhang et al., 2017c; Kardar et al., 2020). In addition, emission wavelength shifts during titration are rare and attractive, which could enhance the detection specificity by adding an additional recognition dimension, in addition to the traditional recognition dimension of fluorescence intensity enhancement or quenching (Hu et al., 2013).

Considering the red-shift factor of fluorescence spectrum, the maximum emission peak corresponding to each titration concentration was selected in the calculation of the quenching rate of antibiotics and the value of fitting curve, instead of the fluorescence emission peak at a fixed wavelength. In this case, when the titrated concentration of NFT and NFZ reached 75 ppm, the fluorescence quenching efficiency rate was 90.9 and 94.1%, respectively. When the titrated concentration of FZD reached 100 ppm, the fluorescence quenching rate was 85.3%. To further understand the luminescent quenching degree, the quenching curves were quantitatively studied by Stern–Volmer equation. As shown in Figure 4, the Stern–Volmer plots illustrated a linear relationship in the low concentration region with a K_SV_ of 0.2212 ppm^−1^ (5.27×10^4^ M^−1^) for NFT, 0.2447 ppm^−1^ (4.85×10^4^ M^−1^) for NFZ, and 0.1660 ppm^−1^ (3.74×10^4^ M^−1^) for FZD. Based on the K_SV_ values, the detection limits of Cu_0.1_/Zn-MOF toward NFT, NFZ, and FZD were calculated to be 95.0 ppb (0.4 μM), 85.8 ppb (0.43 μM), and 126.5 ppb (0.56 μM), respectively.

In general, the Stern–Volmer equation shows a linear relationship between the concentration of the analyte and luminescent strength. However, in the detection of NFT and FZD, the fitting curve shows a double exponential type. In this case, the Stern–Volmer equation should be expressed as I_0_/I-1 = lg[K_SV_]+lg[C]. This means that the quenching of luminescence may be the result of both dynamic and static factors (Yang Y et al., 2020). In addition, fluorescence emissions of NFT, NFZ, FZD, and TC were detected at 257 nm excitation wavelength, and it was found that NFT and FZD had weak fluorescence emission in the 400–510 nm region, while NFZ and TC had almost no fluorescence emission (Supplementary Figure S19). By comparing the fluorescence quenching curves of NFT and FZD titration experiments with the fluorescence emission curves of NFT and FZD itself, it was found that the curve shapes were similar. In other words, at 257 nm excitation wavelength, as the concentration of NFT and FZD increases, the fluorescence spectra are gradually weakened and redshifted, and the coincidence degree with the fluorescence emission peak of NFT and FZD itself gradually increases (Supplementary Figure S20). As a result, at higher concentrations of NFT and FZD, the fluorescence quenching degree was weakened by the fluorescence emission of NFT and FZD itself.

### Luminescent Detection Toward Tetracyclines

**FIGURE 5:**
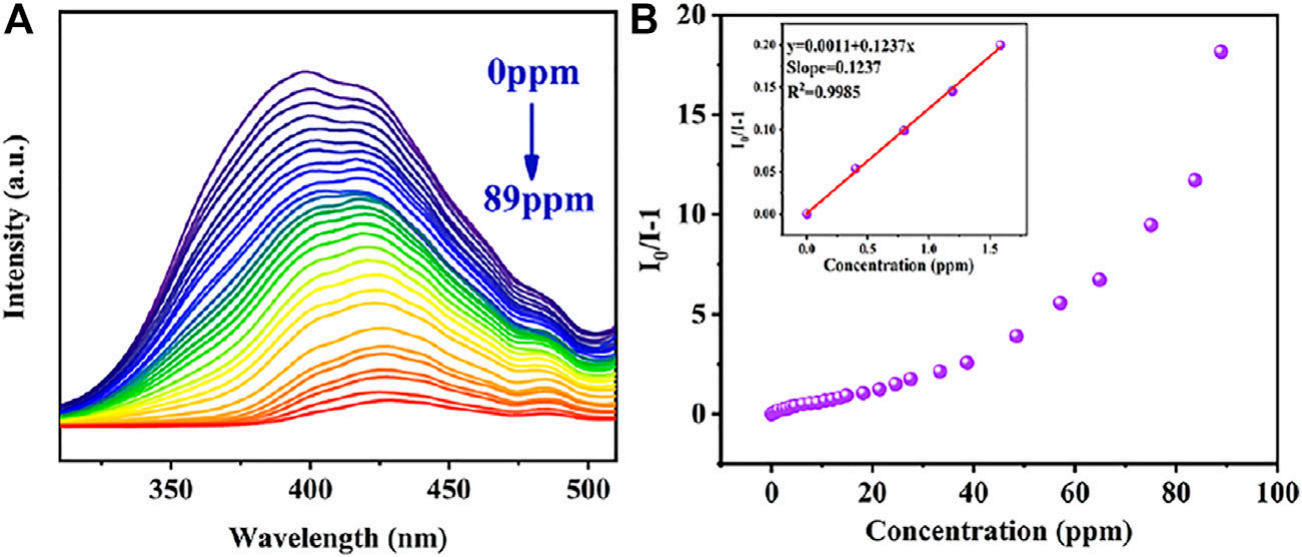
**(A)** Photoluminescence spectra of Cu_0.1_/Zn-MOF in aqueous solutions upon incremental addition of TC. **(B)** Stern–Volmer plot of Cu_0.1_/Zn-MOF upon adding different concentrations of TC.

Similarly, in order to further explore the quantitative detection ability of TC by complex Cu_0.1_/Zn-MOF, the TC solution was dropped into the complex suspension for fluorescence titration (Figure 5A). With the dripping of TC, the fluorescence intensity of the suspension decreases gradually, and the fluorescence quenching occurred when the TC concentration was 89 ppm. The fluorescence quenching rate was 94.8% compared with the initial value. This indicated that Cu_0.1_/Zn-MOF can be used as visual sensor for selective detection of TC in water. The quenching curves were quantitatively studied by Stern–Volmer equation. As shown in Figure 5B, the Stern–Volmer plot illustrated a linear relationship in the low concentration region with a K_SV_ of 0.1237 ppm^−1^ (5.94×10^4^ M^−1^) for TC. Based on the K_SV_ value, the detection limit of Cu_0.1_/Zn-MOF toward TC was calculated to be 169.8 ppb (0.35 μM). Compared with the reported MOF-based sensors for the detection of antibiotics, the result implied that Cu_0.1_/Zn-MOF has potential application value in the detection of NFT, NZF, FZD, and TC (Supplementary Table S4).

### Mechanism Analysis

**FIGURE 6:**
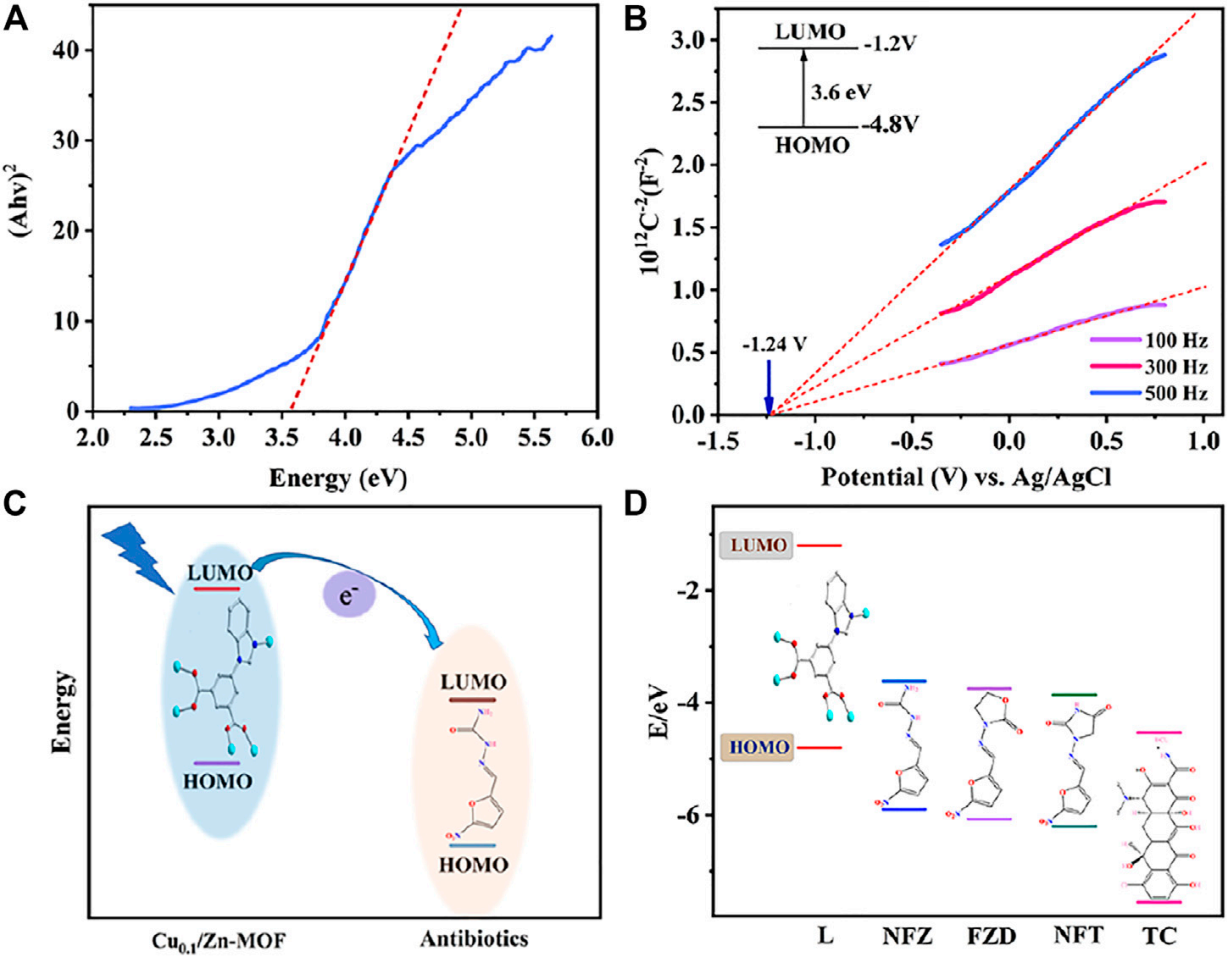
**(A)** Kubelka–Munk plots of Cu_0.1_/Zn-MOF. **(B)** Mott–Schottky plots of Cu_0.1_/Zn-MOF. **(C)** Schematic of electron transfer from the LUMO of MOFs to the LUMO of antibiotics. **(D)** Theoretical HOMO and LUMO energies for selected antibiotics.

The mechanism analysis of antibiotic detection is as follows: the PXRD patterns of Cu_0.1_/Zn-MOF soaked in four antibiotics were basically unchanged compared with those before soaking (Supplementary Figure S9D), which ruled out the possibility of fluorescence quenching caused by skeleton collapse. The excitation spectra of Cu_0.1_/Zn-MOF overlapped with the UV absorption spectra of SM, CHL, CEF, NFT, NFZ, FZD, and TC, but hardly overlapped with other antibiotics (Supplementary Figure S21), indicating that there may be competitive excitation energy absorption effect between the seven antibiotics and Cu_0.1_/Zn-MOF. All the seven antibiotics have fluorescence quenching effect on Cu_0.1_/Zn-MOF, but the fluorescence quenching effect of NFT, NFZ, FZD, and TC is significantly stronger than that of SM, CHL, and CEF, indicating that other factors may be involved in the fluorescence quenching. As shown in Supplementary Figure S21, the emission spectra of Cu_0.1_/Zn-MOF overlapped with the UV absorption spectra of NFT, NFZ, FZD, and TC, and hardly overlapped with other antibiotics. To some extent, this may explain that NFT, NFZ, FZD, and TC have stronger fluorescence quenching rates for Cu_0.1_/Zn-MOF than SM, CHL, and CEF, and suggested that FRET may also be an important reason of fluorescence quenching. In addition, due to the low LUMO energy levels of antibiotics, photoinduced electron transfer (PET) is also a possible luminescence quenching mechanism (Toal and Trogler, 2006; Nagarkar et al., 2014). The valence band energy levels (VB) and conduction band energy levels (CB) of MOF can be described in a pattern similar to molecular orbitals (MOs) (Wang et al., 2016). Specifically, the electrons in the Cu_0.1_/Zn-MOF–occupied orbital (HOMO) are excited to the lowest unoccupied orbital (LUMO), followed by the transfer to the antibiotic with a lower LUMO level. To explain the possibility of this process, we calculated and derived the band structures of Cu_0.1_/Zn-MOF and antibiotics. According to Kubelka–Munk, the bandgap is about 3.6 eV (Figure 6A). As shown in Figure 6B, the Mott−Schottky plots show a flat band potential of about −1.2 V versus Ag/AgCl for Cu_0.1_/Zn-MOF, while the LUMOs of NFT, NFZ, FZD, and TC were calculated to be −3.86 eV, −3.62 eV, −3.75 eV, and −4.53 eV, respectively (Wang et al., 2016; Malik and Iyer, 2017). Due to the conduction band of MOFs being at a higher energy level than the lowest unoccupied molecular orbital (LUMO) of the antibiotic, electrons were transferred from MOFs to the antibiotics (Figures 6C, D), which eventually leads to the fluorescence quenching of MOFs.

### Recyclability and Fast Response Time

The aforementioned experimental results indicated that Cu_0.1_/Zn-MOF displays good water stability and detection sensitivity. In addition, the fluorescence sensor also needs to have good cyclic stability and fast response. After usage, the sensor can be regenerated by centrifugation and acetone washing. After soaking in 5 analytes for 4 h, the main peak of PXRD patterns matched well with that before soaking (Supplementary Figure S9D), and the fluorescence quenching efficiency remained unchanged after 5 cycles (Supplementary Figure S22). The results showed that the sensor exhibited good recyclability. In addition, the sensor showed rapid response to all the five analytes at different concentrations, as shown in Supplementary Figure S22, and the fluorescence intensity decreased within 20 s and remained stable after 120 s.

### Comparison of Detection Sensitivity

It has been proved that the doping of Cu^II^ ions contributes to the improvement of the water stability of **Zn-MOF**. Subsequently, in order to verify the influence of the doping of Cu ions on the sensitivity of detection, the original **Zn-MOF** was used as fluorescence sensors to conduct titration experiments on Fe^3+^, NFT, NFZ, FZD, and TC, respectively. As shown in Supplementary Figure S23–S27, the Stern–Volmer plots illustrated a linear relationship in the low concentration region with a K_SV_ of 1.01×10^4^ M^−1^ for Fe^3+^, 9.33×10^4^ ppm^−1^ (2.22×10^4^ M^−1^) for NFT, 1.12 ×10^5^ ppm^−1^ (2.23×10^4^ M^−1^) for NFZ, 4.74×10^4^ ppm^−1^ (1.07×10^4^ M^−1^) for FZD, and 4.96×10^4^ ppm^−1^ (2.39×10^4^ M^−1^) for TC. The data showed that compared with Cu_0.1_/Zn-MOF, the K_SV_ of **Zn-MOF** for five pollutants are significantly reduced. In other words, proper Cu^II^ doping improves the sensitivity of **Zn-MOF** to detect analytes. The mechanism of the higher sensitivity of Cu_0.1_/Zn-MOF in the detection of analytes is still not completely clear. We speculate that it may be related to the following factors: First, the increase of Stokes shift reduces the background interference, which is beneficial to the strong penetration to the sample, and enhances the detection sensitivity. Second, the liquid UV spectrum displayed that the UV absorption of TC was similar at 257 and 283 nm, while the UV absorptions of Fe^3+^, NFT, NFZ, and FZD were stronger at 257 nm than at 283 nm (Supplementary Figure S28). In other words, it might have stronger excitation competitive absorption at 257 nm, leading to the enhancement of fluorescence quenching.

## Conclusion

In summary, we constructed a **Zn-MOF** complex with superior photophysical property. Subsequently, we successfully doped Cu^II^ ions into **Zn-MOF** in a simple and feasible way to enhance its water stability. This strategy enables LMOFs, which were initially limited by water stability, to implement detection in aqueous solvents. Interestingly, subsequent detection showed that bimetallic LMOFs doped with an appropriate proportion of Cu^II^ ions had lower detection limits for Fe^3+^, NFT, NFZ, FZD, and TC. This study is of great significance to broaden the range of alternative LMOFs in practical application and may provide a new strategy for the design of LMOFs in the future.

## Data Availability

The datasets presented in this study can be found in online repositories. The names of the repository/repositories and accession number(s) can be found below:http://www.ccdc.cam.ac.uk/conts/retrieving.html; The CIF file (CCDC No. 2143524).
