# Supplementary material for: CuII Ion Doping Enhances the Water Stability of Luminescent Metal–Organic Framework, Realizing the Detection of Fe3+ and Antibiotics in Aqueous Solutions
Source: Front Chem. 2022 Feb 28;10:860232. doi: 10.3389/fchem.2022.860232 (PMC8919071; doi:10.3389/fchem.2022.860232)
Supplement: Supplementary file 1 [file DataSheet1.docx]

**Electronic Supplementary Information (ESI)**

**Cu^II^ Ion Doping Enhances the Water Stability of Luminescent Metal-organic Framework, Realizing the Detection of Fe^3+^ and Antibiotics in Aqueous Solutions**

***Ruo-Qin Jia, Geng Tan, Ying-Jun Chen, Lu-Yang Zuo, Bo Li* and Li-Ya Wang****

*College of Chemistry and Pharmacy Engineering, Nanyang Normal University, Nanyang, 473061, P. R. China*

**Corresponding authors:**

*(B. Li) E-mail: [libozzu0107@](mailto:libozzu0107@)163.com

*(L. Y. Wang) E-mail: [wly@nynu.edu.cn](mailto:wly@nynu.edu.cn)

**FT-IR of Cu_x_/Zn-MOF (x=0.01, 0.1, 0.2, 0.5)**

**Cu_0.01_/Zn-MOF**, IR (KBr pellet, cm^–1^): 3458 (w), 3104 (m), 3074 (w), 2289 (w), 2247 (w), 1835 (w), 1787 (w), 1654 (s), 1597 (s), 1510 (s), 1458 (s), 1428 (s), 1390 (s), 1310 (s), 1238 (s), 1179 (m), 1113 (m), 1042 (w), 1003 (w), 921 (s), 847 (w), 786 (s), 749 (s), 716 (s), 689 (m), 650 (w), 586 (w), 530 (m), 456 (s).

**Cu_0.1_/Zn-MOF**, IR (KBr pellet, cm^–1^): 3452 (w), 3104 (m), 3073 (w), 2289 (w), 2247 (w), 1835 (w), 1785 (w), 1651 (s), 1597 (s), 1509 (s), 1459 (s), 1430 (s), 1390 (s), 1310 (s), 1239 (s), 1180 (m), 1113 (m), 1042 (w), 1004 (w), 920 (s), 849 (w), 786 (s), 749 (s), 720 (s), 689 (m), 652 (w), 585 (w), 531 (m), 461 (s).

**Cu_0.2_/Zn-MOF**: IR (KBr pellet, cm^–1^): 3443 (w), 3104 (m), 3074 (w), 2288 (w), 2247 (w), 1834 (w), 1783 (w), 1647 (s), 1598 (s), 1506 (s), 1459 (s), 1430 (s), 1390 (s), 1308 (s), 1237 (s), 1179 (m), 1114 (m), 1041 (w), 1003 (w), 917 (s), 849 (w), 785 (s), 750 (s), 720 (s), 689 (m), 660 (w), 586 (w), 531 (m), 468 (s).

**Cu_0.5_/Zn-MOF**: IR (KBr pellet, cm^–1^): 3445 (w), 3103 (m), 3074 (w), 2288 (w), 2248 (w), 1832 (w), 1778 (w), 1640 (s), 1598 (s), 1502 (s), 1460 (s), 1430 (s), 1389 (s), 1306 (s), 1236 (s), 1180 (m), 1114 (m), 1041 (w), 1003 (w), 914 (s), 850 (w), 785 (s), 750 (s), 724 (s), 688 (m), 660 (w), 584 (w), 532 (m), 478 (s).


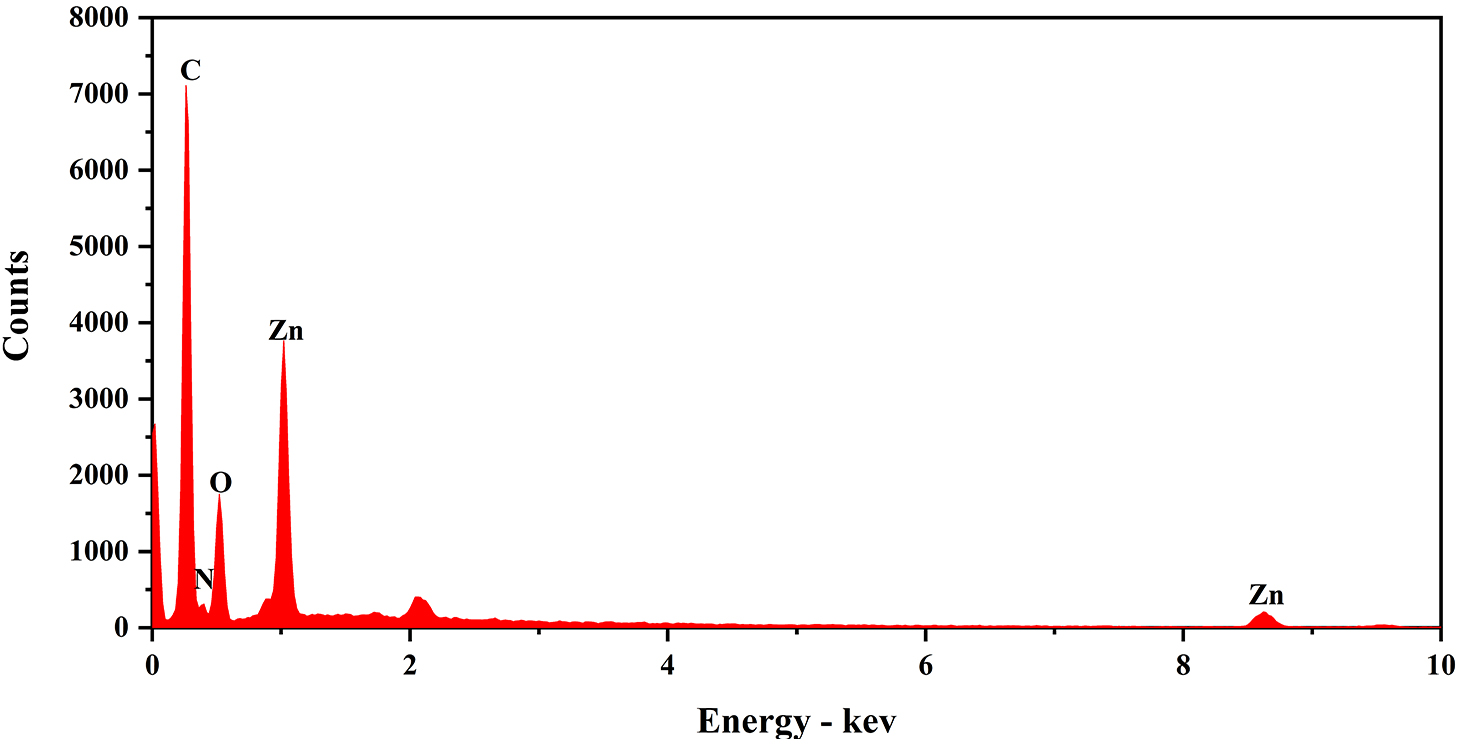


**Figure S1** Energy Dispersive Spectroscopy (EDS) of Zn-MOF.


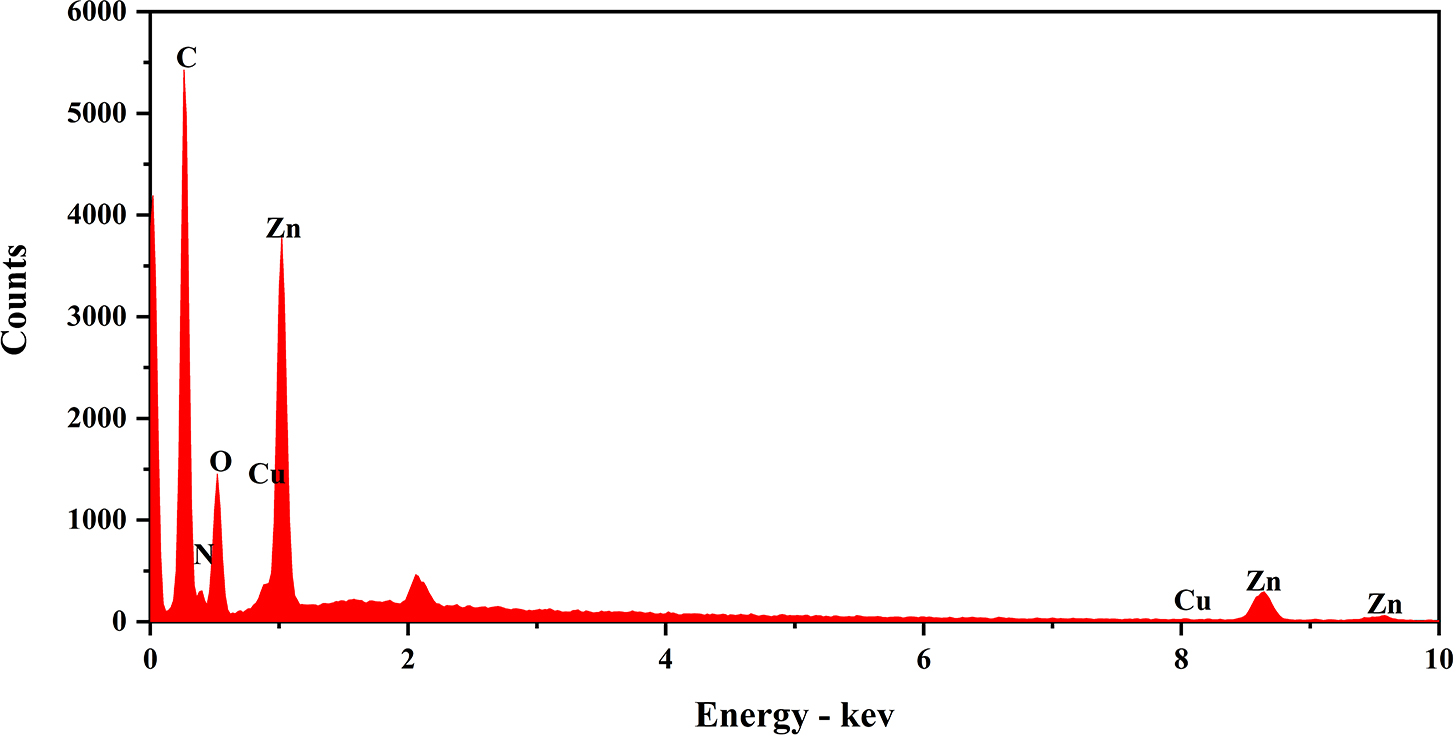


**Figure S2** Energy Dispersive Spectroscopy (EDS) of Cu_0.01_/Zn-MOF.


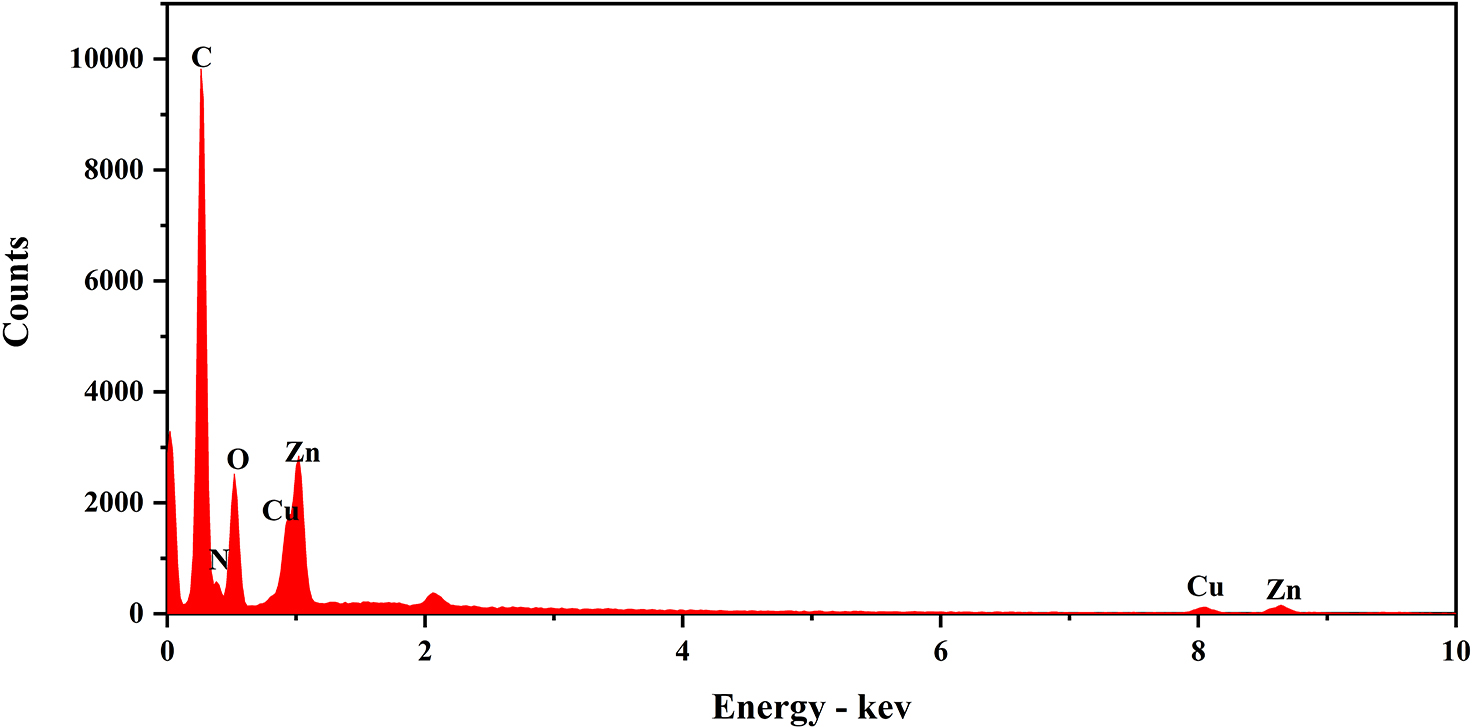


**Figure S3** Energy Dispersive Spectroscopy (EDS) of Cu_0.1_/Zn-MOF.


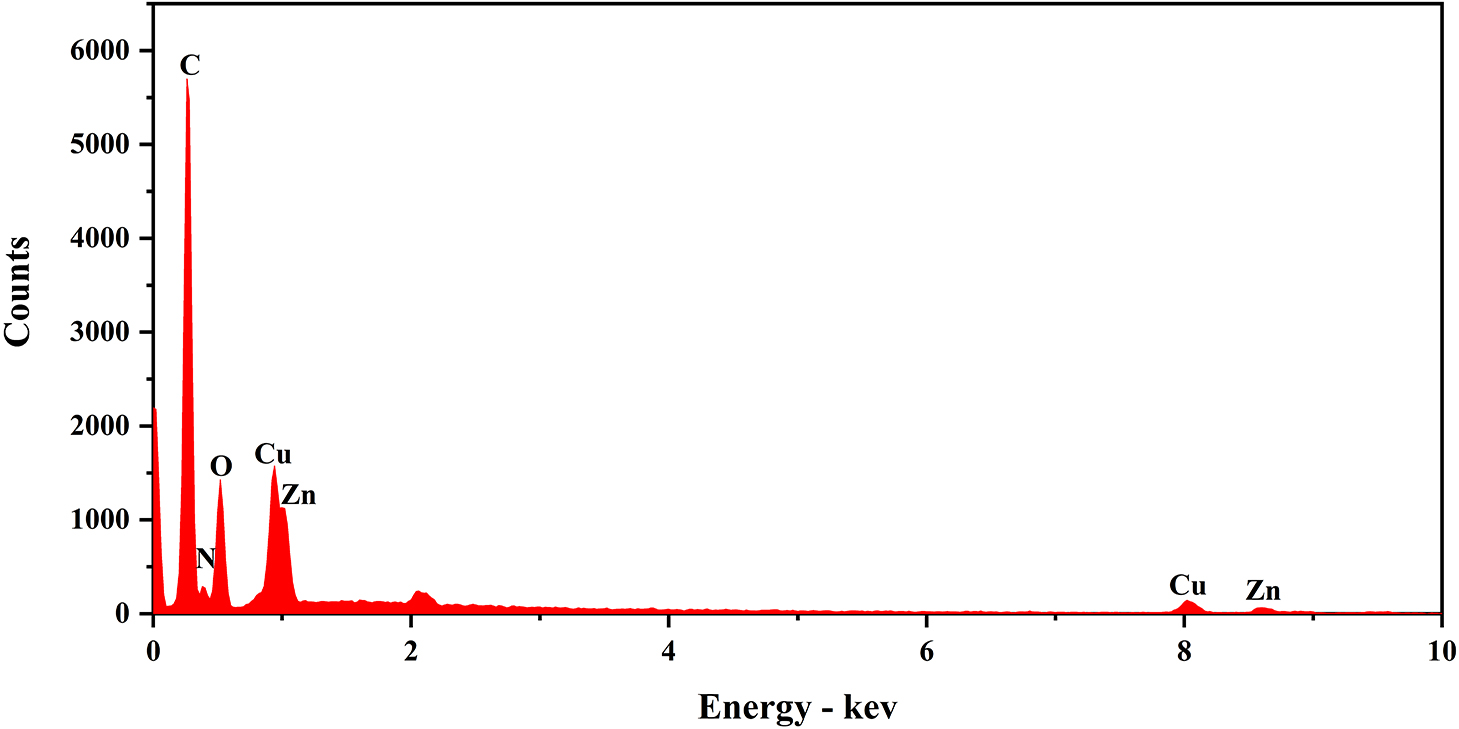


**Figure S4** Energy Dispersive Spectroscopy (EDS) of Cu_0.2_/Zn-MOF.


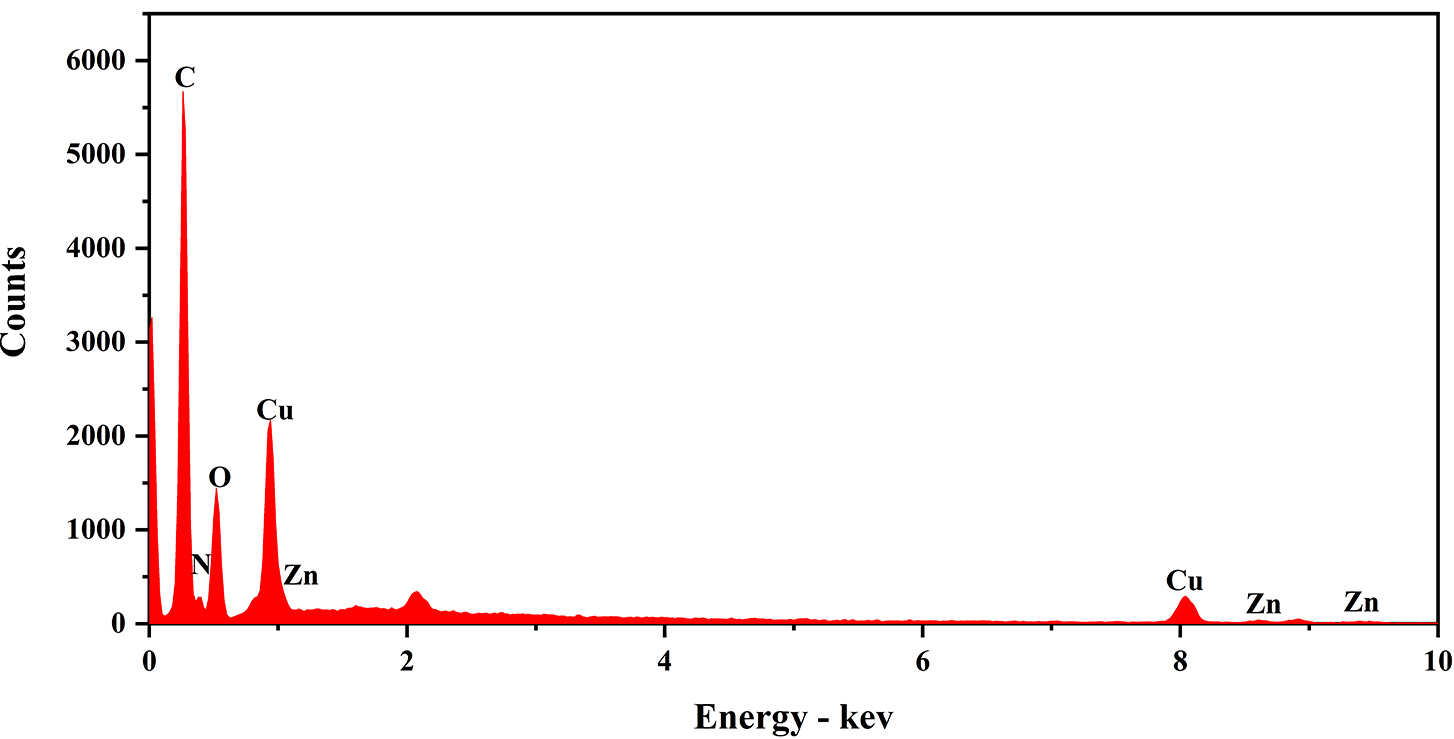


**Figure S5** Energy Dispersive Spectroscopy (EDS) of Cu_0.5_/Zn-MOF.


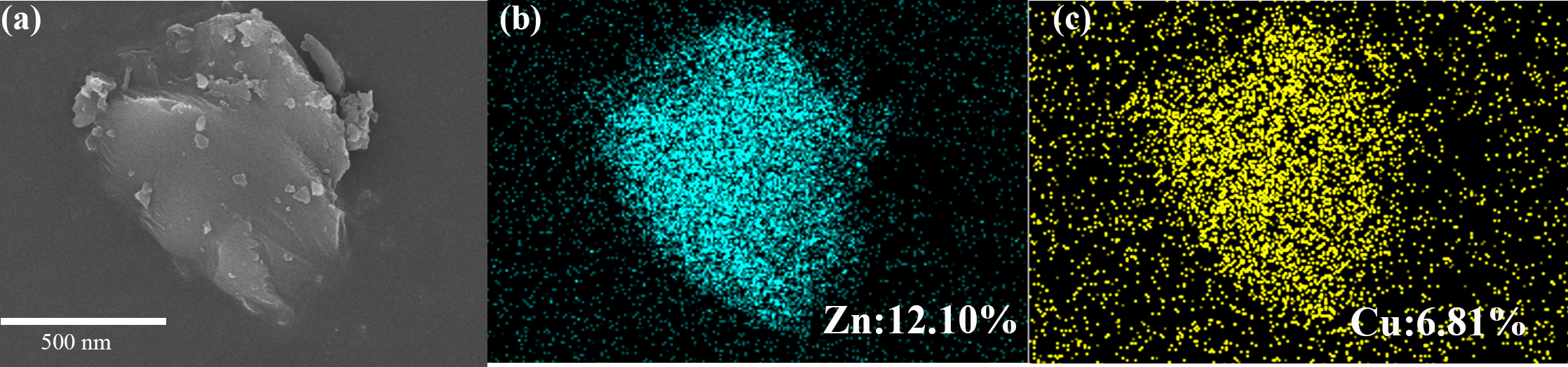


**Figure S6** (a-c) Elemental mapping of Zn (12.10%) and Cu (6.81%).


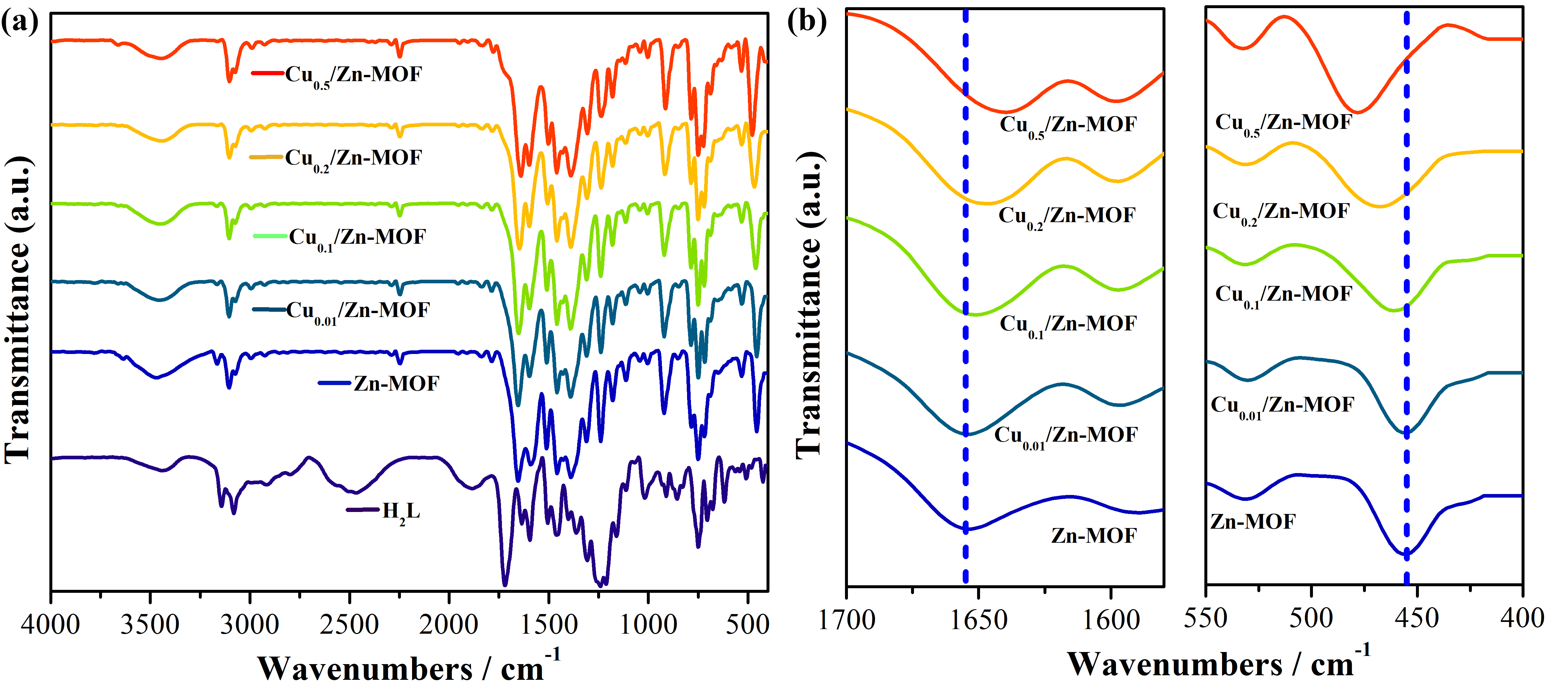


**Figure S7** (a) The FT-IR spectrum of H_2_L, Zn-MOF and Cu_x_/Zn-MOF. (b) FT-IR spectrum shift with copper doping.


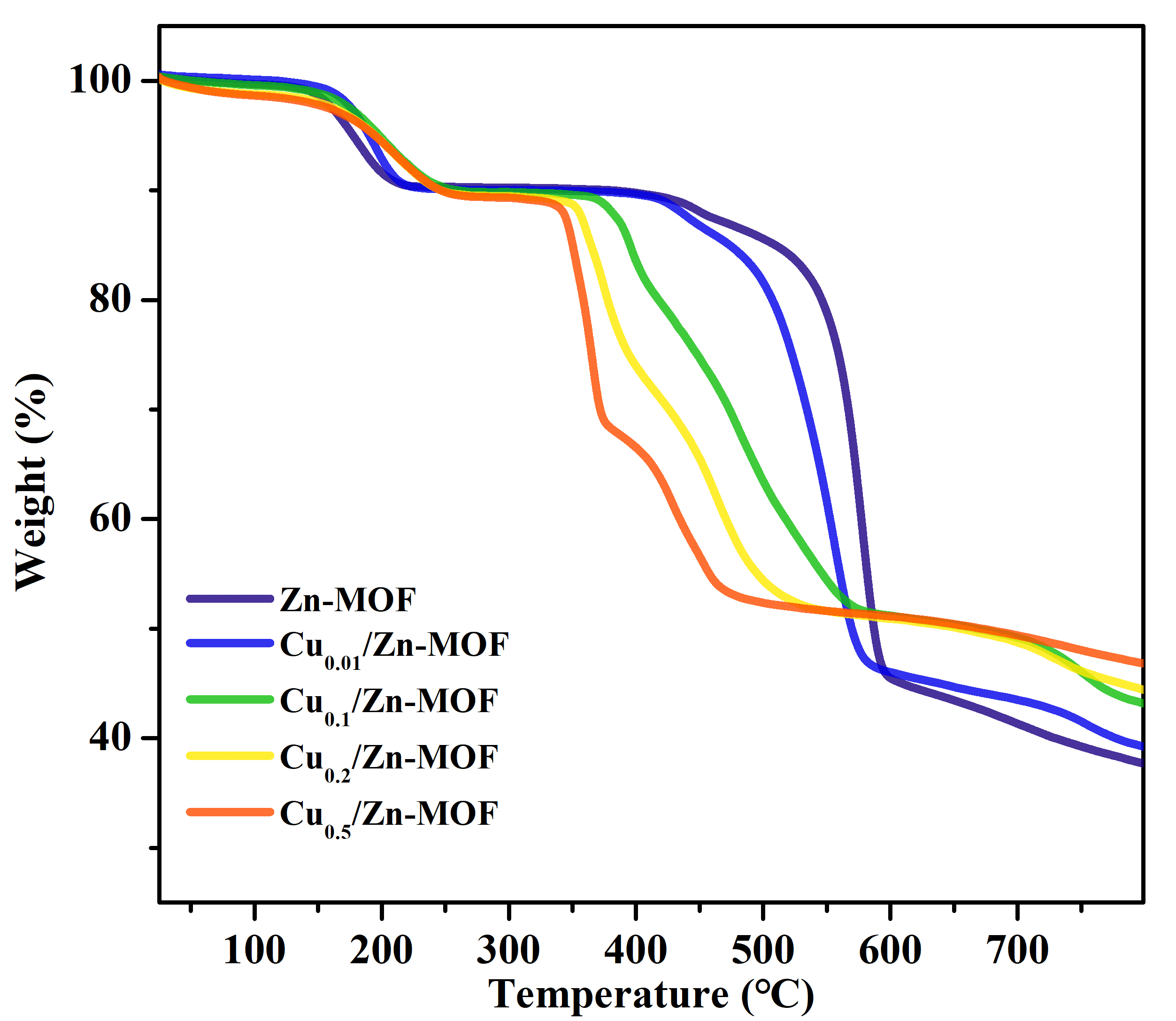


**Figure S8** TGA curves for Zn-MOF and Cu_x_/Zn-MOF.


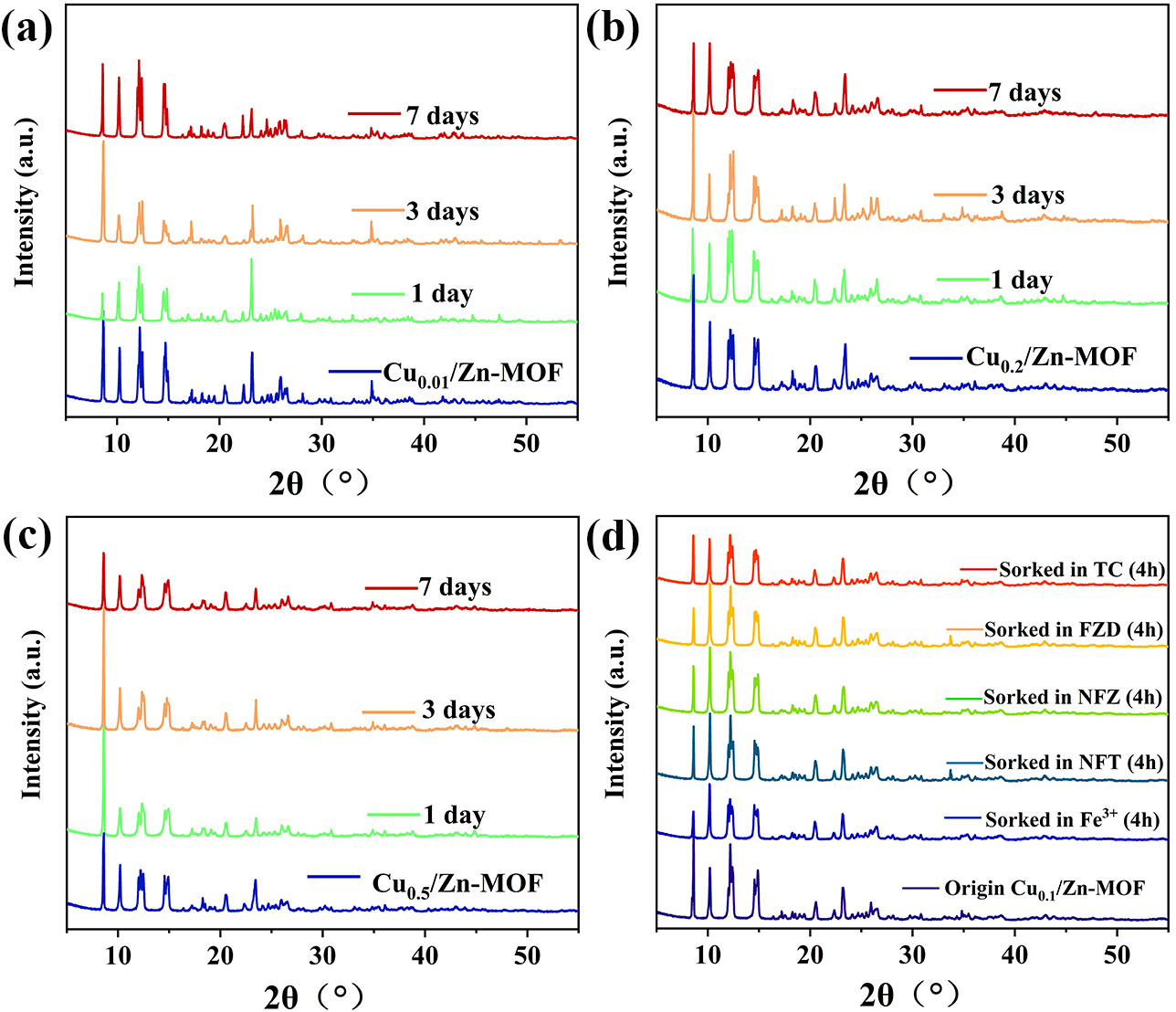


**Figure S9** PXRD patterns of Cu_0.01_/Zn-MOF (a), Cu_0.2_/Zn-MOF (b) and Cu_0.5_/Zn-MOF (c) soaked in water 7 days. (d) PXRD patterns of Cu_0.1_/Zn-MOF soaked in aqueous Fe^3+^, NFT, NFZ, FZD and TC (1 mM) for 4 h.


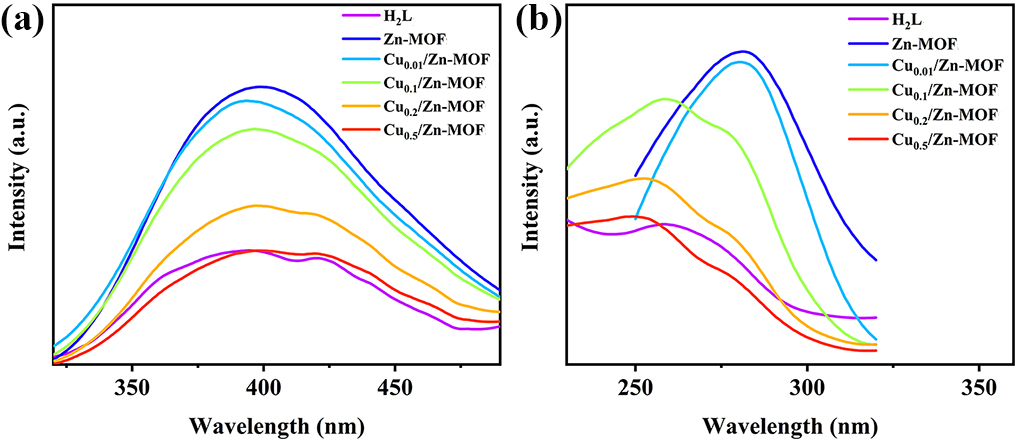


**Figure S10** (a) Emission spectra and (b) Excitation spectra of H_2_L, Zn-MOF and Cu_x_/Zn-MOF in water.


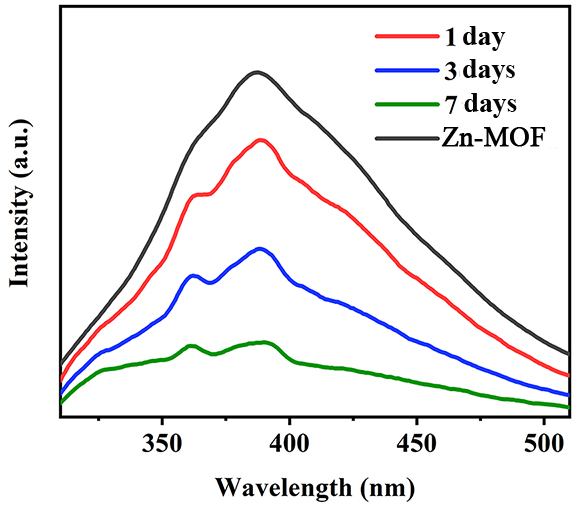


**Figure S11** Emission spectra of Zn-MOF in water and for 1, 3, and 7 days.





**Figure S12** Emission spectra of Cu_0.1_/Zn-MOF in water and for 1, 3, and 7 days.

**
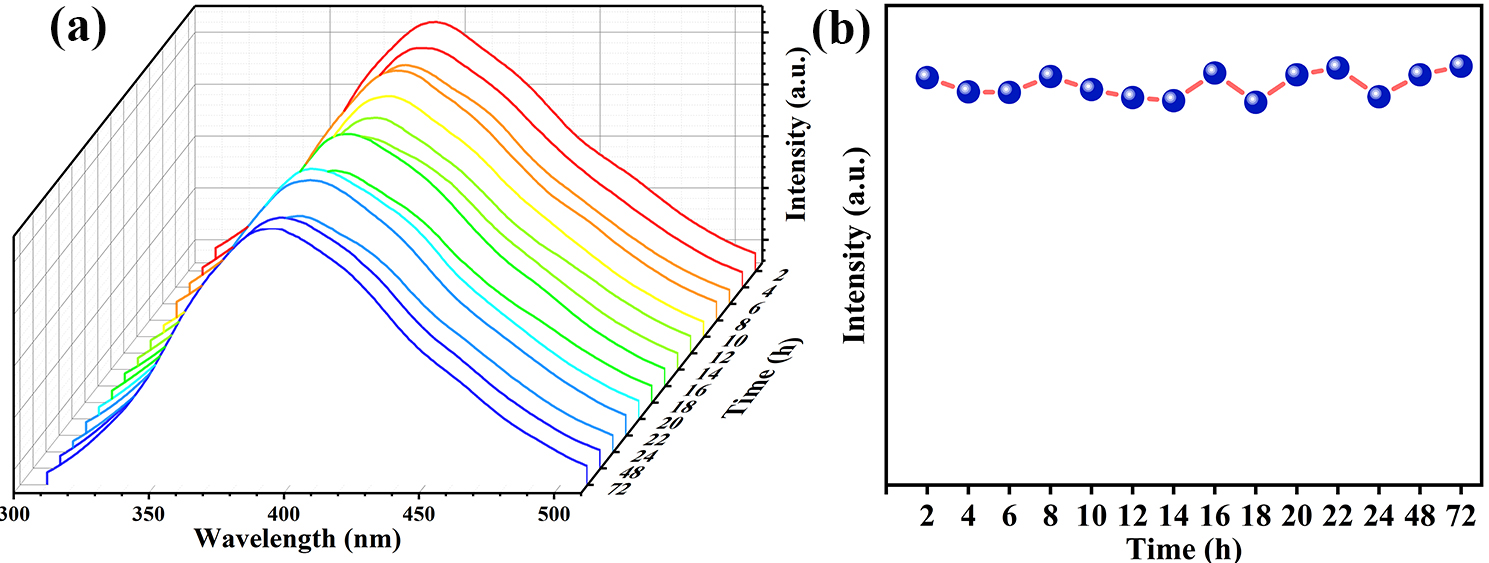
**

**Figure S13** (a, b) Emission spectra of Cu_0.1_/Zn-MOF in water for different time.

^
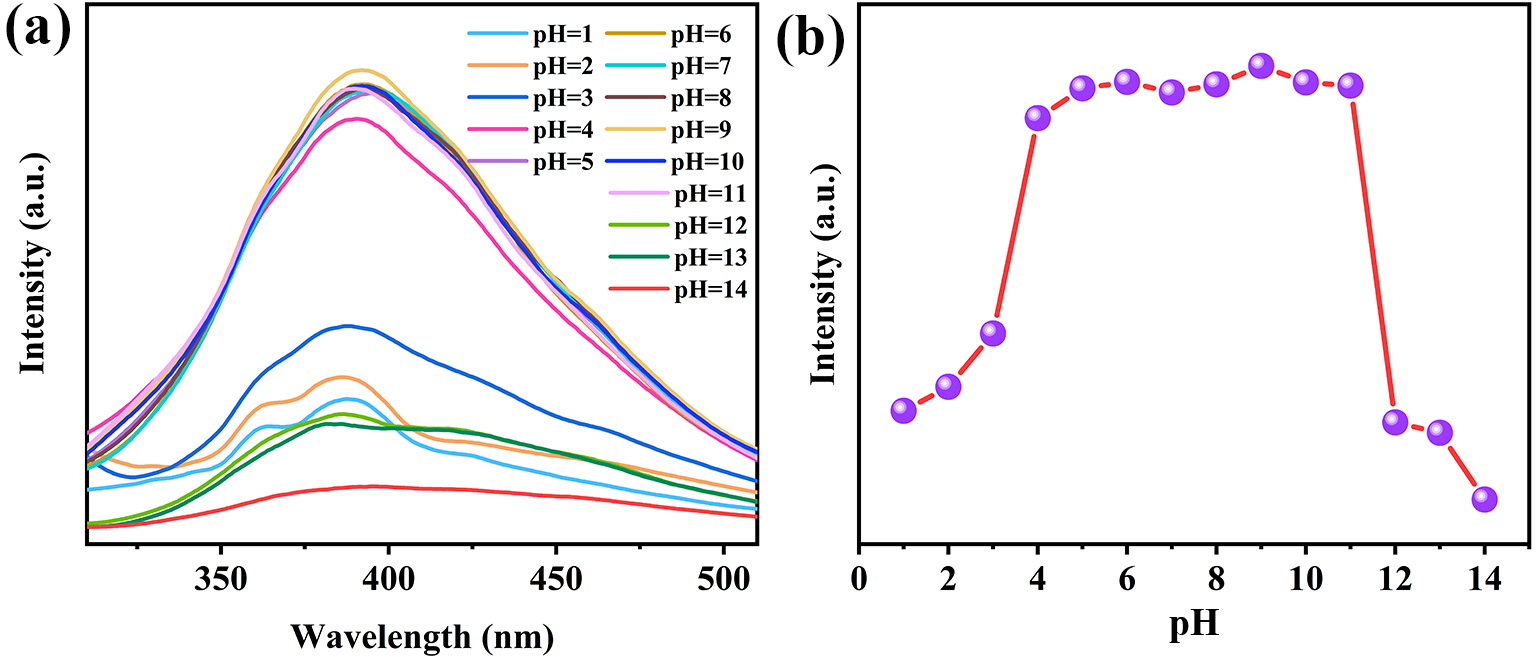
^

**Figure S14** (a, b) Emission spectra of Cu_0.1_/Zn-MOF in aqueous solutions with different pH values (1-14).

^
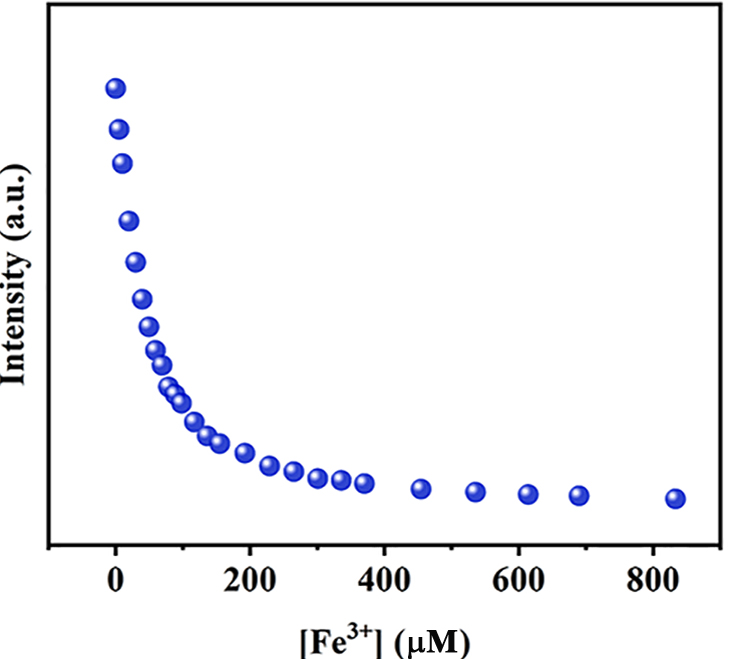
^

**Figure S15** Luminescent intensities of the emission peaks with increasing concentration of Fe^3+^.


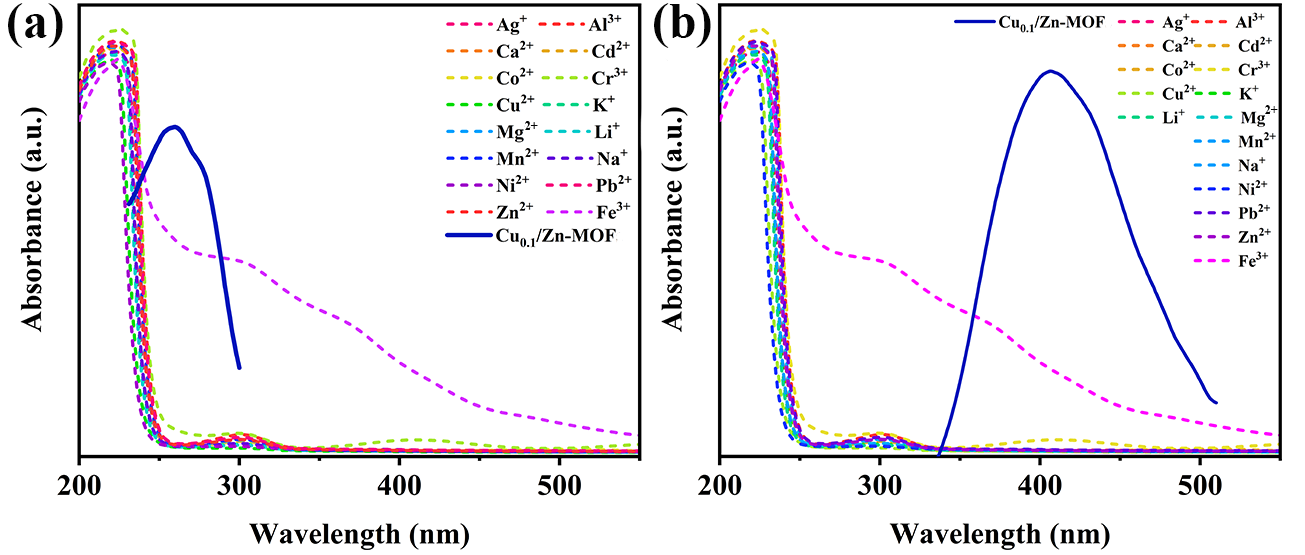


**Figure S16** Spectral overlap of the excitation (b) and emission spectra (b) of Cu_0.1_/Zn-MOF with the absorption spectra of different cations (Na^+^, Ca^2+^, K^+^, Li^+^, Zn^2+^, Mg^2+^, Co^2+^, Mn^2+^, Cd^2+^, Ni^2+^, Ag^+^, Pb^2+^, Cu^2+^, Al^3+^, Cr^3+^, and Fe^3+^).


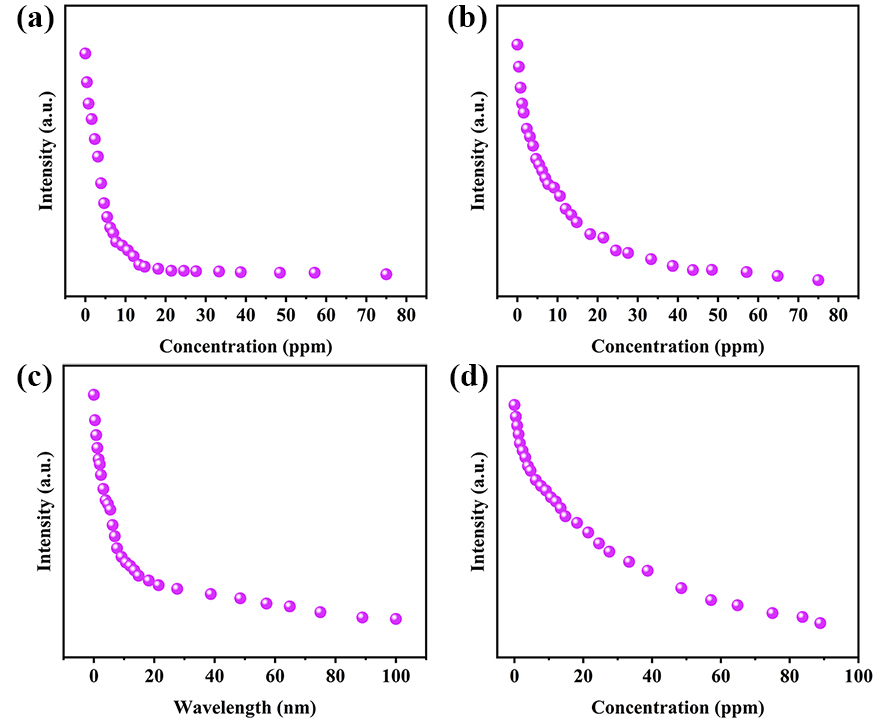


**Figure S17** Luminescent intensities of the emission peaks with increasing concentrations of NFT (a), NFZ (b), FZD (c) and TC (d).


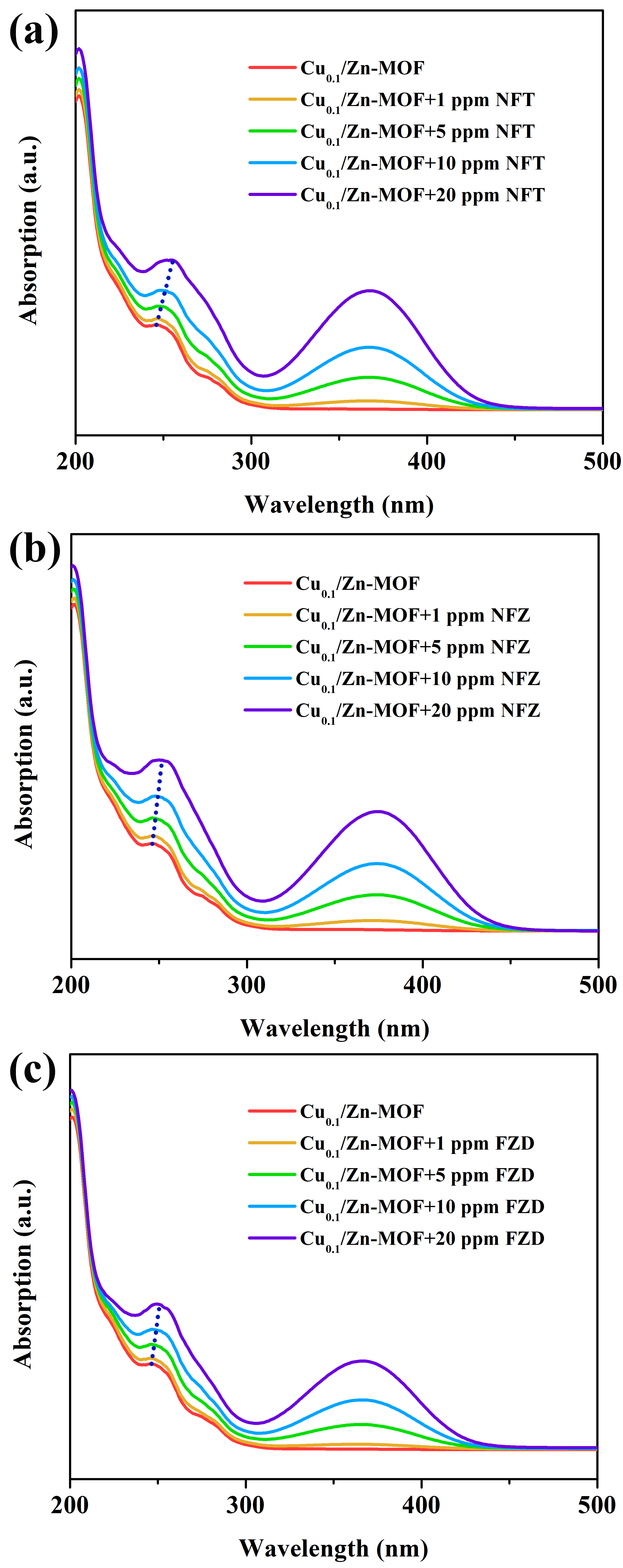


**Figure S18** UV-vis spectra of Cu_0.1_/Zn-MOF before and after NFT (a), NFZ (b) and FZD (c) titration.

^
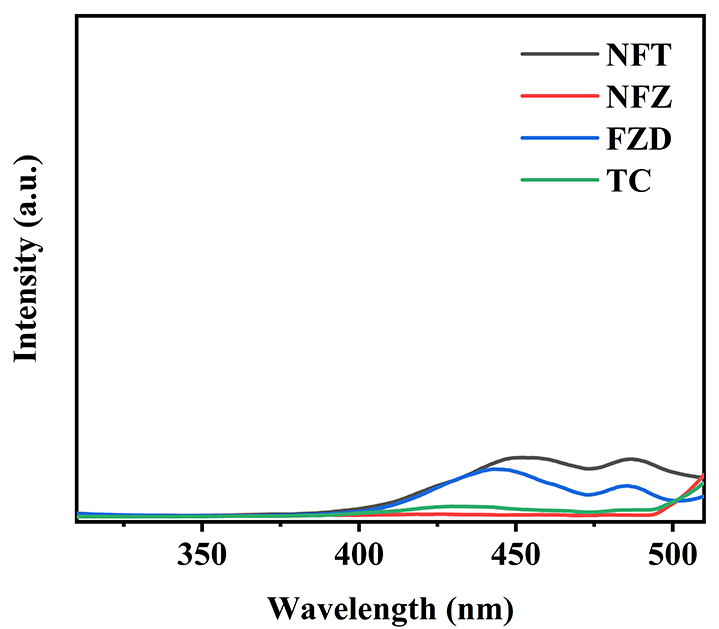
^

**Figure S19** Emission spectra of NFT, NFZ, FZD and TC at 257 nm excitation wavelength.

^
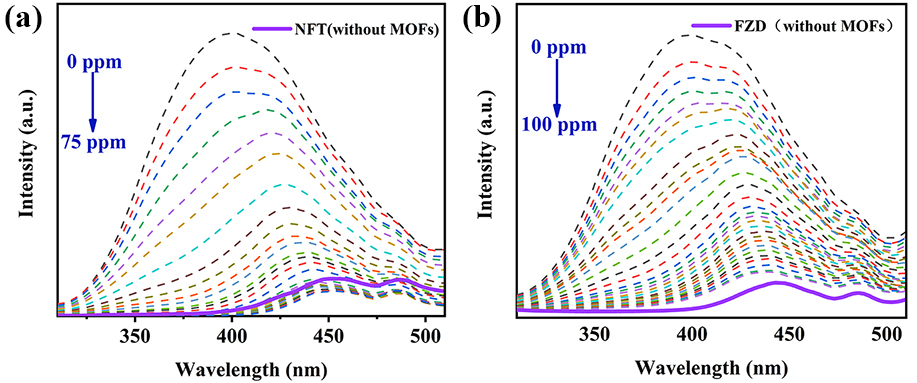
^

**Figure S20** (a) Spectral overlap at 257 nm excitation wavelength of the emission spectra of NFT with the Photoluminescence spectra of Cu_0.1_/Zn-MOF upon incremental addition of NFT. (b) Spectral overlap at 257 nm excitation wavelength of the emission spectra of FZD with the Photoluminescence spectra of Cu_0.1_/Zn-MOF upon incremental addition of FZD.


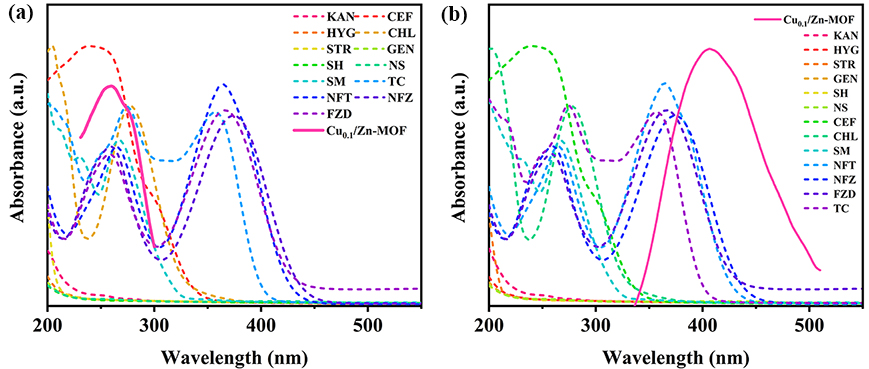


**Figure S21** Spectral overlap of the excitation (a) and emission spectra (b) of Cu_0.1_/Zn-MOF with the absorption spectra of thirteen antibiotics (KAN, HYG, STR, GEN, SH, NS, CEF, CHL, SM, NFT, NFZ, FZD and TC).


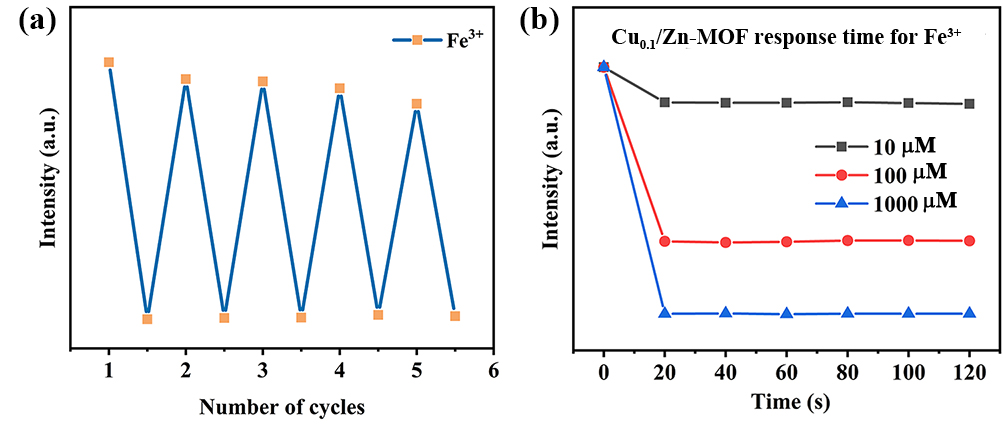


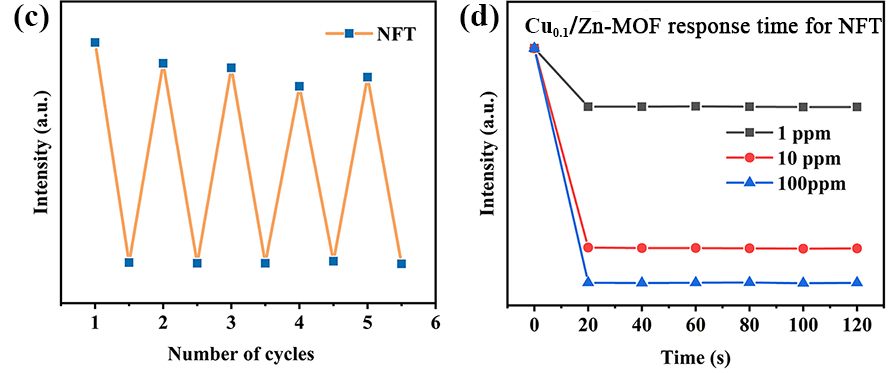


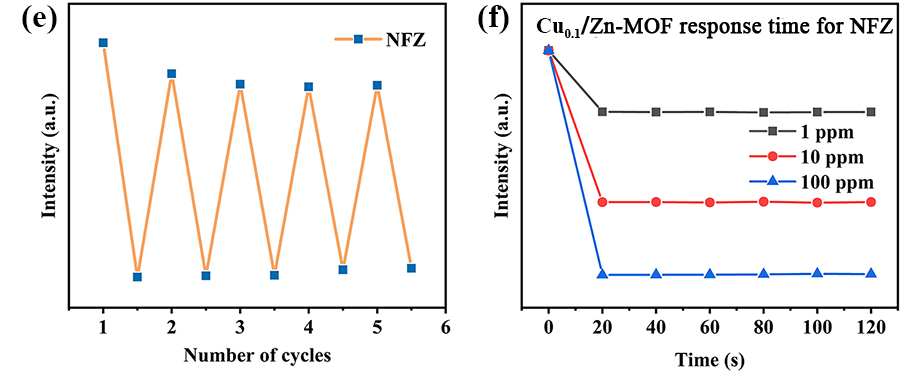


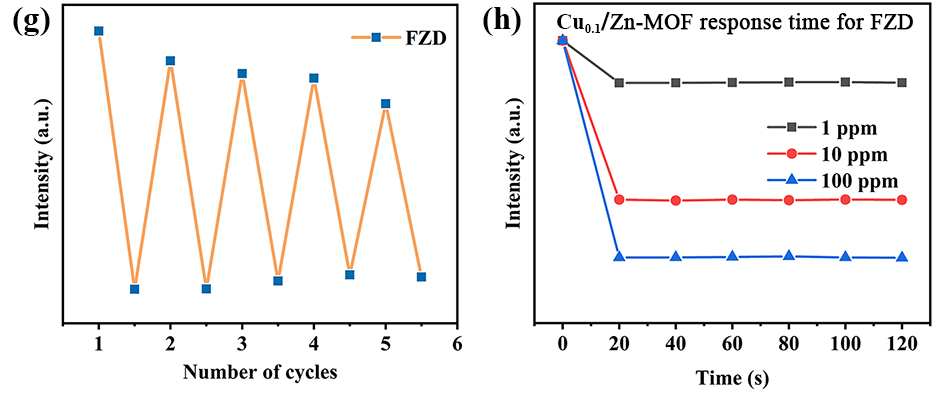


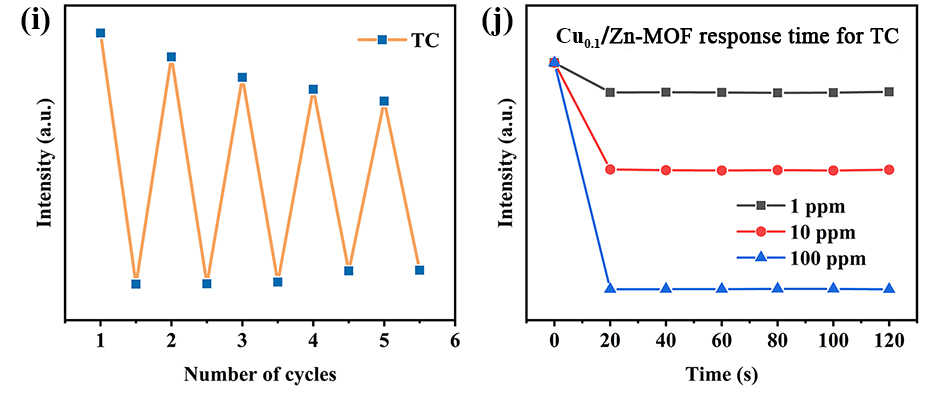


**Figure S22** Recyclability tests of Cu_0.1_/Zn-MOF suspension implemented with Fe^3+^ (a), NFT (c), NFZ (e), FZD (g) and TC (i). Fluorescence response time of Cu_0.1_/Zn-MOF in different concentrations of Fe^3+^ (b), NFT (d), NFZ (f), FZD (h) and TC (j) suspensions, respectively.

^
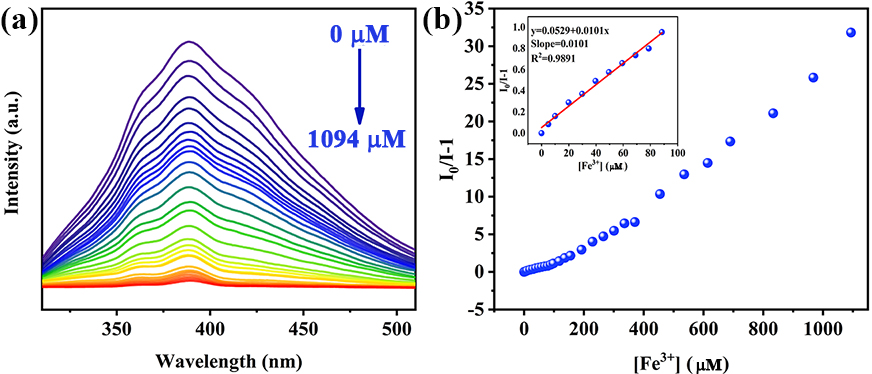
^

**Figure S23** (a) Photoluminescence spectra of Zn-MOF in aqueous solutions containing Fe^3+^. (b) Stern-Volmer plot of Zn-MOF upon adding different concentration of Fe^3+^.


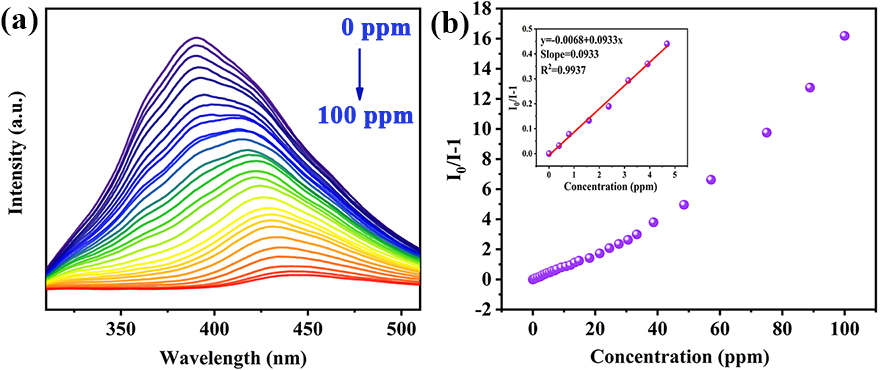


**Figure S24** (a) Photoluminescence spectra of Zn-MOF in aqueous solutions containing NFT. (b) Stern-Volmer plot of Zn-MOF upon adding different concentration of NFT.


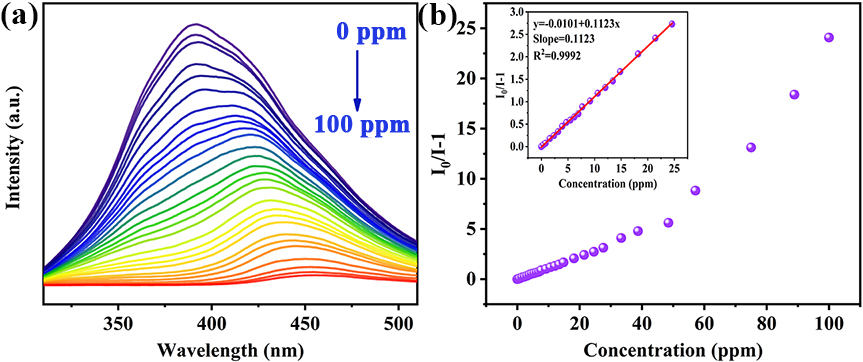


**Figure S25** (a) Photoluminescence spectra of Zn-MOF in aqueous solutions containing NFZ. (b) Stern-Volmer plot of Zn-MOF upon adding different concentration of NFZ.

^
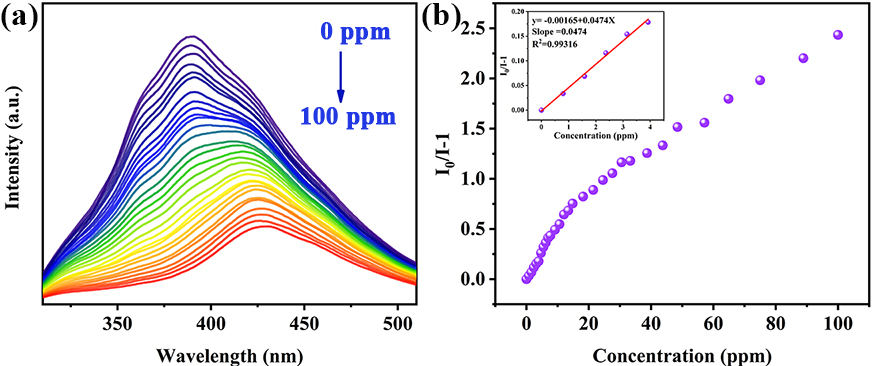
^

**Figure S26** (a) Photoluminescence spectra of Zn-MOF in aqueous solutions containing FZD. (b**)** Stern-Volmer plot of Zn-MOF upon adding different concentration of FZD.


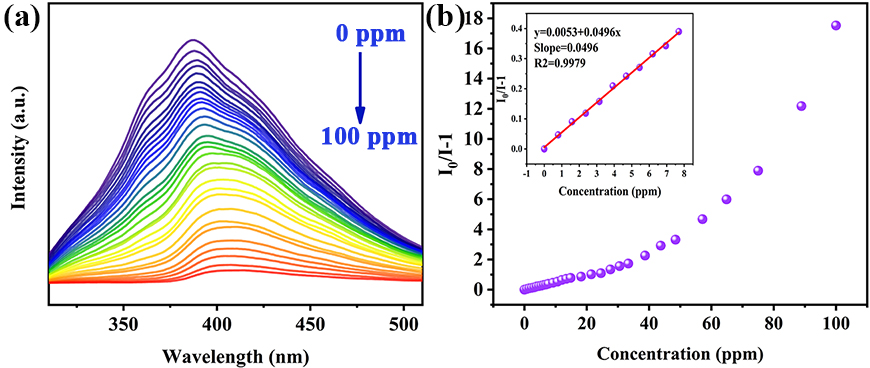


**Figure S27** (a) Photoluminescence spectra of Zn-MOF in aqueous solutions containing TC. (b) Stern-Volmer plot of Zn-MOF upon adding different concentration of TC.


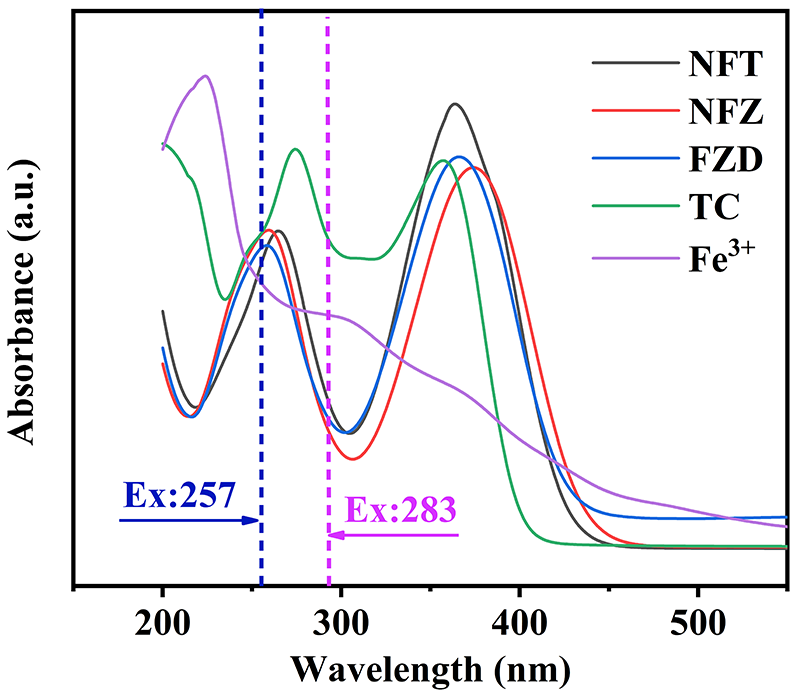


**Figure S28** The UV absorption of five detected substances (Fe^3+^, NFT, NFZ, FZD and TC) was compared at 257nm and 283nm.

**Table S1.** Selected bond lengths (Å) and bond angles (°) for complex **Zn-MOF**.

| **Bond** | **Length Å** | **Bond** | **Length Å** |
| --- | --- | --- | --- |
| O(4)-Zn1#1 | 2.027(2) | Zn1-O(3)#5 | 2.033(2) |
| O(2)-Zn1#2 | 2.030(2) | Zn1-O(1) | 2.035(2) |
| Zn1-N(1)#3 | 2.021(2) | N(1)-Zn1#7 | 2.021(2) |
| Zn1-O(4)#4 | 2.027(2) | O(3)-Zn1#6 | 2.033(2) |
| Zn1-O(2)#2 | 2.030(2) |  |  |
| **Bond** | **Angle °** | **Bond** | **Angle °** |
| N(1)#3-Zn1-O(4)#4 | 96.05(10) | O(4)#4-Zn1-O(1) | 84.91(12) |
| N(1)#3-Zn1-O(2)#2 | 106.07(10) | O(2)#2-Zn1-O(1) | 157.01(11) |
| O(4)#4-Zn1-O(2)#2 | 88.12(12) | O(3)#5-Zn1-O(1) | 87.90(12) |
| N(1)#3-Zn1-O(3)#5 | 106.70(11) | O(4)#4-Zn1-O(3)#5 | 156.78(11) |
| N(1)#3-Zn1-O(1) | 96.45(10) | O(2)#2-Zn1-O(3)#5 | 89.99(11) |
| Symmetry transformations used to generate equivalent atoms: #1 x, -y+1/2, z+1/2; #2 -x+1, -y+1, -z; #3 -x+2, y+1/2, -z+1/2; #4 x, -y+1/2, z-1/2; #5 -x+1, y+1/2, -z+1/2; #6 -x+1, y-1/2, -z+1/2; #7 -x+2, y-1/2, -z+1/2. | | | |

**Table S2** EDS elemental analysis of Zn-MOFs and Cu_x_/Zn-MOF.

| Samples | Element content (%) | | | | | Cu/Zn in reactants | Cu/Zn in products |
| --- | --- | --- | --- | --- | --- | --- | --- |
|  | C | O | N | Zn | Cu |  |  |
| **Zn-MOF** | 59.30 | 16.37 | 4.75 | 19.59 | 0 | -- | -- |
| **Cu_0.01_/Zn-MOF** | 56.68 | 15.24 | 5.17 | 22.59 | 0.31 | 0.010 | 0.014 |
| **Cu_0.1_/Zn-MOF** | 57.16 | 17.09 | 6.84 | 12.10 | 6.81 | 0.108 | 0.563 |
| **Cu_0.2_/Zn-MOF** | 57.36 | 15.91 | 6.36 | 8.44 | 11.93 | 0.243 | 1.412 |
| **Cu_0.5_/Zn-MOF** | 57.08 | 16.54 | 5.04 | 2.66 | 18.68 | 0.972 | 7.023 |

**Table S3** A comparison of various luminescent MOFs probes for the detection of Fe^3+^.

| Materials | solvent | *K*sv(M^-1^) | Detection  limit (μM) | Ref. |
| --- | --- | --- | --- | --- |
| Cu_0.1_/Zn-MOF | water | 2.76 × 10^4^ | 0.76 | This work |
| [Zn_2_(L)_2_(bpe)_2_(H_2_O)_2_] | water | 0.23× 10^4^ | 25 | 1 |
| Zn-MOF | water | 1.326 ×10^4^ | 0.88 | 2 |
| {[Cd_2_ (bptc)(phen)_2_]·4H_2_O}*_n_* | water | 3.07 ×10^3^ | 21.7 | 3 |
| CUST-532 | DMF | 1.01 ×10^4^ | 1.12 | 4 |
| [Eu_2_(MFDA)_2_(HCOO)_2_(H_2_O)_6_]·H_2_O | DMF | 1.58 ×10^3^ | 0.3 | 5 |
| [Zr_6_O_4_(OH)_4_(C_8_H_2_O_4_S_2_)_6_]·DMF·18H_2_O | water | 4.41 ×10^3^ | 1.26 | 6 |
| {[Tb_4_(OH)_4_(DSOA)_2_(H_2_O)_8_].(H_2_O)_8_}*_n_* | water | 3.543×10^4^ | -- | 7 |

**Table S4** A comparison of various luminescent MOFs probes for the detection of NFT, NZF, FZD and TC.

| Materials | antibiotics | solvent | *K*sv (M^-1^) | Detection  limit (μM) | Ref. |
| --- | --- | --- | --- | --- | --- |
| Cu_0.1_/Zn-MOFs | NFT | water | 5.27×10^4^ | 0.4 | This work |
| Cu_0.1_/Zn-MOFs | NFZ | water | 4.85×10^4^ | 0.43 | This work |
| Cu_0.1_/Zn-MOFs | FZD | water | 3.74×10^4^ | 0.56 | This work |
| Cu_0.1_/Zn-MOFs | TC | water | 5.94×10^4^ | 0.35 | This work |
| [Cd_2_(L)(bpda)_2_]·3DMF·H_2_O | FZD | water | 1.1×10^4^ | 4.3 | 8 |
| [Eu_2_Na(Hpdbb)(pddb)_2_(CH_3_COO)_2_]·2.5DMA}*_n_* | FZD | DMF | 2.13×10^4^ | 1.06 | 9 |
| Cd-CBCD | NFT | water | 6.4×10^4^ | 0.55 | 10 |
| In-MOF | NFT | water | 6.3×10^4^ | 30 | 11 |
| Dy-TCPB | FZD | water | 6.6×10^4^ | 0.05 | 12 |
| HNU-52 | NFZ | DMF | -- | 0.72 | 13 |
| [Cd_2_Na(L)(BDC)_2.5_]·9H_2_O | NFZ | DMF | -- | 0.82 | 14 |
|  | NFT | DMF | -- | 1.15 |  |
|  | FZD | DMF | -- | 2.19 |  |
| PCN-128Y | TC | water | 9.84×10^5^ | 0.03 | 15 |
| In-sbdc | TC | water | -- | 0.28 | 16 |

**Table S5** A comparison of Zn-MOF and Cu_0.1_/Zn-MOF for the detection of Fe^3+^, NFT, NZF, FZD and TC.

| Detected objects | Materials | *K*sv(M^-1^) |
| --- | --- | --- |
| Fe^3+^ | Zn-MOF | 1.01× 10^4^ |
|  | Cu_0.1_/Zn-MOF | 2.76 × 10^4^ |
| NFT | Zn-MOF | 2.22 × 10^4^ |
|  | Cu_0.1_/Zn-MOF | 5.27×10^4^ |
| NFZ | Zn-MOF | 2.23×10^4^ |
|  | Cu_0.1_/Zn-MOF | 4.85×10^4^ |
| FZD | Zn-MOF | 1.07×10^4^ |
|  | Cu_0.1_/Zn-MOF | 3.74×10^4^ |
| TC | Zn-MOF | 2.39×10^4^ |
|  | Cu_0.1_/Zn-MOF | 5.94×10^4^ |

**References**

1. Hu F. L., Shi Y. X., Chen H. H., Lang J. P. (2015). A Zn (II) coordination polymer and its photocycloaddition product: syntheses, structures, selective luminescence sensing of iron (III) ions and selective absorption of dyes. Dalton Trans. 44, 18795-18803.
2. Rath B. B., Vittal J. J. (2020). Water stable Zn (II) metal–organic framework as a selective and sensitive luminescent probe for Fe (III) and chromate ions. Inorg. Chem. 59, 8818-8826.
3. Lin Y., Zhang X., Chen W., Shi W., Cheng P. (2017). Three cadmium coordination polymers with carboxylate and pyridine mixed ligands: luminescent sensors for FeIII and CrVI ions in an aqueous medium. Inorg. Chem. 56, 11768-11778.
4. Fan M., Sun B., Li X., Pan Q., Sun J., Ma P., Su Z. (2021). Highly Fluorescent Cadmium Based Metal–Organic Frameworks for Rapid Detection of Antibiotic Residues, Fe^3+^ and Cr_2_O_7_^2–^ Ions. Inorg. Chem. 60, 9148-9156.
5. Zhou X. H., Li L., Li H. H., Li A., Yang T., Huang W. (2013). A flexible Eu (III)-based metal–organic framework: turn-off luminescent sensor for the detection of Fe (III) and picric acid. Dalton Trans. 42, 12403-12409.
6. Dalapati R., Kökçam-Demir Ü., Janiak C., Biswas S. (2018). The effect of functional groups in the aqueous-phase selective sensing of Fe (III) ions by thienothiophene-based zirconium metal–organic frameworks and the design of molecular logic gates. Dalton Trans. 47, 1159-1170.
7. Dong X. Y., Wang R., Wang J. Z., Zang S. Q., Mak T. C. W. (2015). Highly selective Fe3+ sensing and proton conduction in a water-stable sulfonate–carboxylate Tb–organic-framework. J. Mater. Chem. A 3, 641-647.
8. Xu Y. L., Liu Y., Liu X. H., Zhao Y., Wang P., Wang Z. L., Sun W. Y. (2018). Novel cadmium(II) frameworks with mixed carboxylate and imidazole-containing ligands for selective detection of antibiotics. Polyhedron 154, 350-356.
9. Xu S., Shi J. J., Ding B., Liu Z. Y., Wang X. G. Zhao X. J., Yang E. C. (2019). A heterometallic sodium (i)–europium (iii)-organic layer exhibiting dual-responsive luminescent sensing for nitrofuran antibiotics, Cr_2_O_7_^2−^ and MnO_4_^−^ anions. Dalton Trans. 48, 1823-1834.
10. Xu N., Zhang Q., Zhang G. (2019). A carbazole-functionalized metal–organic framework for efficient detection of antibiotics, pesticides and nitroaromatic compounds. Dalton Trans. 48, 2683-2691.
11. Li Y. Z., Wang G. D., Lu Y. K., Hou L., Wang Y. Y., Zhu Z. (2020). A multi-functional in (iii)-organic framework for acetylene separation, carbon dioxide utilization, and antibiotic detection in water. Inorg. Chem. 59, 15302-15311.
12. Wu S., Zhu M., Zhang Y., Kosinova M., Fedin V. P., Gao E. (2020). A Water‐Stable Lanthanide Coordination Polymer as Multicenter Platform for Ratiometric Luminescent Sensing Antibiotics. Chem. Eur. J. 26, 3137-3144.
13. Yang Y., Ren G., Yang W., Qin X., Gu D., Liang Z., Guo D. Y., Pan Q. H. (2021). A new MOF-based fluorescent sensor for the detection of nitrofuran antibiotics. Polyhedron 194, 114923.
14. Zhao D., Liu X. H., Zhao Y., Wang P., Liu Y., Azam M., Al-Resayes S. I., Lu Y., Sun W. Y. (2017). Luminescent Cd (II)–organic frameworks with chelating NH_2_ sites for selective detection of Fe (III) and antibiotics. J. Mater. Chem. A 5 (30), 15797-15807.
15. Zhou Y., Yang Q., Zhang D., Gan N., Li Q., Cuan J. (2018). Detection and removal of antibiotic tetracycline in water with a highly stable luminescent MOF. Sensors and Actuat. B: Chem. 262, 137-143.
16. Liu Q., Ning D., Li W. J., Du X. M., Wang Q., Li Y., Ruan W. J. (2019). Metal–organic framework-based fluorescent sensing of tetracycline-type antibiotics applicable to environmental and food analysis. Analyst, 144, 1916-1922.
